# The association between early pregnancy maternal lipid indicators with gestational diabetes mellitus and pre-eclampsia

**DOI:** 10.1016/j.jlr.2025.100942

**Published:** 2025-11-11

**Authors:** Jiapeng Tang, Ye Chen, Mengting Sun, Manjun Luo, Yige Chen, Kebin Chen, Qi Zou, Yuan Peng, Tingting Wang, Jiabi Qin

**Affiliations:** 1 Department of Epidemiology and Health Statistics, Xiangya School of Public Health, Central South University, Changsha, Hunan, China; 2 Department of Science and Education, Xiangya Changde Hospital, Changde, China; 3 Department of Epidemiology and Health Statistics, School of Public Health, Kunming Medical University, Kunming, China

**Keywords:** gestational diabetes mellitus, pre-eclampsia, lipid profiles, lipid-derived indicators, cohort, early pregnancy

## Abstract

The relationship between early pregnancy lipid indicators and gestational diabetes mellitus (GDM) or pre-eclampsia (PE) remains incompletely elucidated. This prospective cohort study explored the associations between seven lipid indicators and GDM and PE among 32,411 pregnant participants. The results suggested that triglycerides (TGs), total cholesterols (TCs), and remnant cholesterols (RCs) were positively associated with both GDM and the composite outcome (GDM/PE). For GDM, compared with the lowest quartile, the highest quartile had odds ratios (ORs) of 1.646 (95% confidence interval [95% CI]: 1.363, 1.988) for TGs, 1.654 (95% CI: 1.241, 2.205) for TCs, and 1.396 (95% CI: 1.189, 1.640) for RCs. For GDM/PE, the corresponding ORs in the highest versus lowest quartile were 1.564 (95% CI: 1.302, 1.877) for TGs, 1.655 (95% CI: 1.253, 2.186) for TCs, and 1.379 (95% CI: 1.180, 1.612) for RCs. Non-HDL-C showed a negative association with GDM and GDM/PE, with ORs of 0.833 (95% CI: 0.758, 0.916) and 0.871 (95% CI: 0.795, 0.954), respectively. TG/HDL-C ratio was positively associated with PE, with an OR of 2.451 (95% CI: 1.369, 4.388). The OR values of the second and third quantiles of HDL-C for PE were 1.706 (95% CI: 1.301, 2.238) and 1.598 (95% CI: 1.170, 2.183), respectively. Nonlinear dose-response relationships were observed for most lipids with the outcomes. Additionally, early pregnancy TG, RC, and TG/HDL-C ratio partially mediated the effect of maternal age on all three outcomes (mediated proportion 2–7%). Non-HDL-C mediated the age-PE pathway (1%). This study simultaneously included multiple lipid parameters for systematic analysis, revealing the impact of dyslipidemia on pregnancy outcomes from a more comprehensive perspective and providing richer evidence for exploring related mechanisms and clinical assessment.

Gestational diabetes mellitus (GDM) and pre-eclampsia (PE) are common pregnancy complications worldwide, endangering maternal and fetal health. GDM refers to any degree of glucose intolerance that occurs or is detected first during pregnancy. GDM had a 2021 global prevalence of 14% (95% confidence interval [95% CI]: 13.97, 14.04) (1). In mainland China, the prevalence was approximately 14.8% (95% CI: 12.8, 16.7) (2). GDM links to adverse perinatal outcomes and future health risks for mothers and offspring (3, 4, 5, 6). PE, with new onset hypertension and proteinuria after 20 weeks of gestation, has a global prevalence of 2–4% (7). In China, PE reported an approximate prevalence of 2.74% (8). It also causes adverse perinatal outcomes and raises long-term maternal health risks (9, 10, 11, 12). Given the substantial health burden imposed by GDM and PE, identifying modifiable risk factors is critical for developing preventive strategies.

GDM and PE are affected by multiple factors, such as maternal age, prepregnancy overweight or obesity, and smoking during pregnancy (13, 14, 15). Emerging evidence highlights the association of maternal lipid levels with GDM and PE. Meta-analyses demonstrated significantly elevated lipid levels in GDM/PE patients compared with normal pregnant women (16, 17), and cohort studies further linked early pregnancy high triglycerides (TGs) to increased GDM/PE risk (18, 19). Mendelian randomization studies identified TG and HDL-C as mediators of the association between obesity and GDM (20) and found that increased HDL-C reduced the incidence of PE and GDM (21). Other lipid markers such as total cholesterol (TC) and LDL-C also relate to GDM/PE risk (22, 23).

Besides the basic lipid indicators mentioned above, some lipid-derived indicators that are derived from basic lipid indicators by formula calculation have also attracted much attention, such as remnant cholesterol (RC), non-HDL-C, and TG/HDL-C ratio. Existing studies suggest that these lipid-derived indicators may be superior to basic lipid indicators and provide a more comprehensive picture of lipid levels and lipid metabolism (24). RC is defined as the cholesterol content of remnant lipoproteins generated during the intravascular remodeling of triglyceride-rich lipoproteins (25). High RC levels have been widely recognized as strongly associated with increased risk of all-cause mortality, cardiovascular disease, and type 2 diabetes mellitus (26, 27, 28). Currently, some studies suggest that early pregnancy RC is positively associated with GDM risk (29, 30, 31). Non-HDL-C refers to the TC content of all lipoprotein particles that carried proatherogenic potential, including LDL, TG-rich lipoproteins (VLDL, IDL, and chylomicrons), and lipoprotein (a) (32). Some studies suggest that non-HDL-C is an independent risk factor for GDM in patients with polycystic ovary syndrome (33). However, some studies have found no significant difference in non-HDL-C levels between GDM and non-GDM populations (34). Notably, higher prepregnancy non-HDL-C levels were significantly associated with increased risks of PE (35). Furthermore, elevated non-HDL-C during early pregnancy showed positive correlations with both antenatal blood pressure and long-term blood pressure measurements at 6 and 9 years postpartum (36). TG/HDL-C ratio is an important indicator for assessing insulin resistance (IR) and cardiovascular-metabolic risk (37). Although several studies have reported that TG/HDL-C ratio is a valid predictor of GDM (23, 38), some studies failed to identify statistically significant differences in TG/HDL-C ratio between GDM and non-GDM populations (39). In conclusion, while individual lipid indicators have been extensively studied, there are limited studies that comprehensively evaluate the association between various lipid indicators and GDM in early pregnancy, and the role of lipid-derived indicators in GDM remains to be fully explored. In addition, previous studies have explored the association between dyslipidemia and PE, but the vast majority of the results were found at high TG levels and mainly focused on the third trimester of pregnancy. Therefore, this study, based on a large early pregnancy cohort in central China, aims to validate the associations between maternal lipid profiles and lipid-derived indicators in early pregnancy and both GDM and PE, to provide a scientific basis for the reduction of GDM and PE incidence and for early prevention.

## Materials and methods

### Study design and participants

The present study was a prospective cohort study. To construct a large early pregnancy cohort, a total of 39,214 women with spontaneous pregnancies were consecutively recruited into this cohort from March 13, 2013 to December 31, 2019. All participants received their first antenatal care at the Hunan Provincial Maternal and Child Health Care Hospital and planned to continue antenatal care and delivery at the same hospital. Written informed consent was obtained from all participants. For this analysis, we excluded participants who met any of the following criteria: *1*) had multiple pregnancies; *2*) were lost to follow-up; *3*) had termination of pregnancy due to artificial abortion or induced labor; *4*) had diabetes or hypertension before pregnancy; *5*) had missing laboratory data; *6*) unable to obtain an outcome diagnosis at the end of follow-up or unable to cooperate in completing the survey due to severe mental disorder; *7*) had inadequate fasting (<8 h) prior to blood collection. Ultimately, a total of 32,411 pregnant women were included in this analysis (Supplemental Fig. S1). The study was approved by the Ethics Committee of Xiangya School of Public Health, Central South University (approval number: XYGW2018-36) and registered with the Chinese Clinical Trials Registry (registration number: ChiCTR1800016635). This study strictly adhered to the ethical principles of the Declaration of Helsinki set forth by the World Medical Association.

### Data collection

To ensure the accuracy and consistency of data collection, we conducted strict and uniform training for the investigators before the study began. After participants were recruited, the investigators conducted a face-to-face survey using a self-designed structured questionnaire to collect the sociodemographic characteristics and various prepregnancy exposure information. Investigators followed up participants in this cohort to collect data on maternal outcomes and biochemical indicators. All disease diagnosis data and examination results were obtained by querying the electronic medical record system and conducting follow-up visits.

### Lipid measurements

Blood samples after fasting for at least 8 h were collected among all participants during early pregnancy (<14 weeks) to measure serum lipid levels (TG, TC, HDL-C, and LDL-C). A commercial enzymatic assay (Roche Diagnostics, Mannheim, Germany) and a Cobas c702 analyzer were used to measure serum lipid levels. RC (mmol/l) was calculated as TC (mmol/l) minus LDL-C (mmol/l) minus HDL-C (mmol/l). Non-HDL-C (mmol/l) was calculated as TC (mmol/l) minus HDL-C (mmol/l). The TG/HDL-C ratio was calculated by dividing TG (mmol/l) by HDL-C (mmol/l). Based on previous studies (30, 40), participants were stratified into quartiles (Q1, Q2, Q3, and Q4) based on maternal lipid profiles and lipid-derived indicators, with Q1 designated as the reference group.

### Ascertainment of outcomes

The outcomes of interest in this study were GDM and PE, which were retrieved from the electronic medical record system. GDM was diagnosed by conducting a 75 g oral glucose tolerance test in pregnant women at 24–28 weeks. The diagnostic criteria followed the International Association of Diabetes and Pregnancy Study Groups criteria (41), which states that the diagnosis of GDM is made when any one of the following three glucose levels is reached or exceeded: fasting plasma glucose >5.10 mmol/l, 1-h plasma glucose >10.00 mmol/l, and 2-h postprandial plasma glucose >8.50 mmol/l. The plasma glucose levels were measured using an automatic analyzer (Toshiba TBA-120FR, Tokyo, Japan) in the Central Laboratory of Hunan Maternal and Child Health Hospital. PE was diagnosed based on standardized clinical criteria (42), defined as new-onset hypertension (systolic blood pressure ≥140 mm Hg and/or diastolic blood pressure ≥90 mm Hg, confirmed twice ≥4 h apart) after 20 weeks of gestation, along with either significant proteinuria (≥300 mg/24-h urine, protein-to-creatinine ratio ≥0.3, or random urine protein ≥1+ if quantitative testing was unavailable) or evidence of end-organ dysfunction (hepatic, renal, hematologic, neurologic, or cardiopulmonary impairment) in the absence of proteinuria. Additionally, the composite diagnosis of GDM and/or PE was included as an additional outcome in this study.

### Covariates

Confounding factors in relation to outcomes and lipid levels were considered as covariates in this study. Based on previous studies (30, 31) and the data we collected, we selected several confounders as follows: maternal age (<35 or ≥35 years), ethnicity (Han or minority), residence location (urban areas or rural areas), education level (junior high school or below, senior middle school, college, master or above), monthly household income (≤2,500, 2,500–5,000, or >5,000¥), drinking before pregnancy (yes or no), smoking before pregnancy (yes or no), drinking in early pregnancy (yes or no), smoking in early pregnancy (yes or no), parity (primipara or multipara), family history of diabetes (yes or no), family history of hypertension (yes or no), and maternal prepregnancy BMI (prepregnancy BMI). Maternal prepregnancy BMI was calculated as prepregnancy weight (kg) divided by height squared (m^2^) and categorized into four groups according to the criteria for Chinese adults: underweight (<18.5 kg/m^2^), normal weight (18.5–23.9 kg/m^2^), overweight (24.0–27.9 kg/m^2^), and obesity (≥28.0 kg/m^2^).

### Statistical analysis

In this study, EpiData, version 3.1 (EpiData Association, Odense, Denmark) was used to establish the database by double entry. In the description of the basic characteristics of the participants, categorical variables were described in the form of a number (proportion), whereas continuous variables that are approximately normally distributed were described in the form of mean ± standard deviation. Intergroup comparisons were performed using Chi-square tests for categorical variables and a *t-*test for continuous variables. The prevalence of outcomes with their 95% CIs was calculated based on an approximation of the binomial distribution to the normal distribution. The logistic regression model was used to estimate the OR and 95% CI*s* between maternal lipid profiles (TG, TC, HDL-C, and LDL-C) or lipid-derived indicators (RC, non-HDL-C, and TG/HDL-C ratio) and outcomes (GDM, PE, and GDM/PE). Sensitivity analyses were conducted by adjusting for different covariates in multivariate models. Model 1 was not adjusted for any covariates. Model 2 was adjusted for maternal age, residence location, education, ethnicity, monthly household income, maternal prepregnancy BMI, drinking before pregnancy, smoking before pregnancy, drinking in early pregnancy, smoking in early pregnancy, parity, family history of diabetes, and family history of hyperglycemia. Model 3 was further adjusted for other lipid profiles and lipid-derived indicators, except for the grouping variables based on model 2. Tests for linear trends were performed by using the quartile median value of each quartile as a quasi-continuous variable in the model (43). Subgroup analyses were conducted using covariates demonstrating significant interactions with lipid profiles or lipid-derived indicators to further evaluate the robustness of the associations between lipid profiles or lipid-derived indicators and risks of outcomes. Restricted cubic spline (RCS) regression was conducted to address the potential nonlinearity of the association between lipid profiles or lipid-derived indicators and outcomes. In addition, mediation analysis was also conducted in this study. Mediation analysis aims to identify and verify the mediation effect of the mediator variable between the independent variable and the dependent variable, that is, the independent variable can indirectly affect the outcome variable by partially or completely changing the level of the mediator variable. In this study, mediation analysis was used to explore the potential mediating effects of basic lipid indicators and extended lipid indicators in the association between maternal age and outcome variables. The mediation package in R software was used, and the nonparametric bootstrap method (with 1,000 resamples) was adopted to estimate the CI of the mediating effect. All the above analyses were conducted using SPSS version 26.0 (IBM Corporation, Armonk, NY) software and R version 4.2.3 (R Foundation for Statistical Computing, Vienna, Austria). Two-sided tests were used for this study, and a *P* value <0.05 was considered statistically significant, unless otherwise stated.

## Results

### Characteristics of participants

A total of 32,411 pregnant women were included in this study with a mean maternal age of 31.05 ± 4.49 years. The characteristics of the population classified by three different outcomes (GDM, PE, and GDM/PE) are shown in Table 1. Statistically significant differences in characteristics of maternal age, education level, prepregnancy maternal BMI, parity, family history of hypertension, lipid profiles (TG, TC, HDL-C, and LDL-C), and lipid-derived indicators (RC, non-HDL-C, and TG/HDL-C ratio) were all observed between the two groups when stratified by any of the three outcomes (*P* < 0.05), whereas residence location was only significant when grouped with both GDM and PE outcomes, drinking before pregnancy was only significant when grouped with both PE and GDM/PE outcomes, and monthly household income was only significant when grouped with PE. In the total study population, the incidence of GDM was 15.37% (95% CI: 14.98, 15.77), the incidence of PE was 1.86% (95% CI: 1.71, 2.00), and the incidence of GDM/PE was 16.89% (95% CI: 16.48, 17.30). Supplemental Table S1 presents the incidence and 95% CI of the three outcomes across quartiles of lipid profiles and lipid-derived indicators.

**Table 1:** Baseline characteristics of participants by different outcomes

Characteristics	Total (%)	GDM (%)			PE (%)			GDM/PE (%)		
		Yes	No	*P*	Yes	No	*P*	Yes	No	*P*
No. of subjects	32,411	4,983	27,428		602	31,809		5,475	26,936	
Maternal age (years)	31.05 ± 4.49	32.37 ± 4.49	30.81 ± 4.45	**<0.001**	31.86 ± 4.92	31.04 ± 4.48	**<0.001**	32.20 ± 4.54	30.80 ± 4.43	**<0.001**
Maternal age (years)				**<0.001**			**<0.001**			**<0.001**
<35	25,313 (78.1)	3,431 (68.9)	21,882 (79.8)		404 (67.1)	24,909 (78.3)		3,764 (68.7)	21,549 (80.0)	
≥35	7,098 (21.9)	1,552 (31.1)	5,546 (20.2)		198 (32.9)	6,900 (21.7)		1,711 (31.3)	5,387 (20.0)	
Ethnicity				0.541			0.569			0.158
Han	32,005 (98.7)	4,925 (98.8)	27,080 (98.7)		596 (99.0)	31,409 (98.7)		5,417 (98.9)	26,588 (98.7)	
Minority	406 (1.3)	58 (1.2)	348 (1.3)		6 (1.0)	400 (1.3)		58 (1.1)	348 (1.3)	
Residence location				**0.009**			**<0.001**			0.482
Urban areas	20,080 (62.0)	3,170 (63.6)	16,910 (61.7)		300 (49.8)	19,780 (62.2)		3,415 (62.4)	16,665 (61.9)	
Rural areas	12,331 (38.0)	1,813 (36.4)	10,518 (38.3)		302 (50.2)	12,029 (37.8)		2,060 (37.6)	10,271 (38.1)	
Educational level				**<0.001**			**<0.001**			**<0.001**
Junior high school or below	2,457 (7.6)	389 (7.8)	2,068 (7.5)		120 (19.9)	2,337 (7.3)		504 (9.2)	1,953 (7.3)	
Senior middle school	9,159 (28.3)	1,281 (25.7)	7,878 (28.7)		217 (36.0)	8,942 (28.1)		1,468 (26.8)	7,691 (28.6)	
College	14,786 (45.6)	2,418 (48.5)	12,368 (45.1)		187 (31.1)	14,599 (45.9)		2,556 (46.7)	12,230 (45.4)	
Master or above	6,009 (18.5)	895 (18.0)	5,114 (18.6)		78 (13.0)	5,931 (18.6)		947 (17.3)	5,062 (18.8)	
Monthly household income (¥)				0.794			**0.021**			0.923
≤2,500	5,605 (17.3)	877 (17.6)	4,728 (17.2)		90 (15.0)	5,515 (17.3)		952 (17.4)	4,653 (17.3)	
2,500–5,000	17,299 (53.4)	2,642 (53.0)	14,657 (53.4)		355 (59.0)	16,944 (53.3)		2,929 (53.5)	14,370 (53.3)	
>5,000	9,507 (29.3)	1,464 (29.4)	8,043 (29.3)		157 (26.1)	9,350 (29.4)		1,594 (29.1)	7,913 (29.4)	
Prepregnancy maternal BMI				**<0.001**			**<0.001**			**<0.001**
Normal weight	22,856 (70.5)	3,406 (68.4)	19,450 (70.9)		332 (55.1)	22,524 (70.8)		3,697 (67.5)	19,159 (71.1)	
Underweight	4,725 (14.6)	426 (8.5)	4,299 (15.7)		53 (8.8)	4,672 (14.7)		463 (8.5)	4,262 (15.8)	
Overweight	4,029 (12.4)	899 (18.0)	3,130 (11.4)		140 (23.3)	3,889 (12.2)		1,014 (18.5)	3,015 (11.2)	
Obesity	801 (2.5)	252 (5.1)	549 (2.0)		77 (12.8)	724 (2.3)		301 (5.5)	500 (1.9)	
Parity				**<0.001**			**0.019**			**<0.001**
Primipara	10,036 (31.0)	1,383 (27.8)	8,653 (31.5)		160 (26.6)	9,876 (31.0)		1,510 (27.6)	8,526 (31.7)	
Multipara	22,375 (69.0)	3,600 (72.2)	18,775 (68.5)		442 (73.4)	21,933 (69.0)		3,965 (72.4)	18,410 (68.3)	
Smoking before pregnancy (yes)	330 (1.0)	46 (0.9)	284 (1.0)	0.468	11 (1.8)	319 (1.0)	**0.046**	55 (1.0)	275 (1.0)	0.912
Drinking before pregnancy (yes)	557 (1.7)	91 (1.8)	466 (1.7)	0.525	22 (3.7)	535 (1.7)	**<0.001**	112 (2.0)	445 (1.7)	**0.041**
Smoking in early pregnancy (yes)	430 (1.3)	70 (1.4)	360 (1.3)	0.601	9 (1.5)	421 (1.3)	0.716	78 (1.4)	352 (1.3)	0.487
Drinking in early pregnancy (yes)	471 (1.5)	81 (1.6)	390 (1.4)	0.269	14 (2.3)	457 (1.4)	0.071	93 (1.7)	378 (1.4)	0.096
Family history of diabetes (yes)	445 (1.4)	136 (2.7)	309 (1.1)	**<0.001**	10 (1.7)	435 (1.4)	0.540	146 (2.7)	299 (1.1)	**<0.001**
Family history of hyperglycemia (yes)	802 (2.5)	187 (3.8)	615 (2.2)	**<0.001**	34 (5.6)	768 (2.4)	**<0.001**	211 (3.9)	591 (2.2)	**<0.001**
TG (mmol/l)	3.63 ± 1.23	3.89 ± 1.30	3.58 ± 1.21	**<0.001**	3.80 ± 1.15	3.63 ± 1.23	**0.001**	3.88 ± 1.29	3.58 ± 1.21	**<0.001**
TC (mmol/l)	6.44 ± 1.14	6.40 ± 1.20	6.45 ± 1.13	**0.002**	6.37 ± 1.17	6.44 ± 1.14	0.119	6.39 ± 1.20	6.45 ± 1.13	**<0.001**
HDL-C (mmol/l)	1.75 ± 0.33	1.73 ± 0.33	1.75 ± 0.33	**<0.001**	1.69 ± 0.34	1.75 ± 0.33	**<0.001**	1.72 ± 0.33	1.75 ± 0.33	**<0.001**
LDL-C (mmol/l)	3.67 ± 0.76	3.61 ± 0.78	3.68 ± 0.76	**<0.001**	3.61 ± 0.72	3.67 ± 0.76	0.055	3.60 ± 0.77	3.68 ± 0.76	**<0.001**
RC (mmol/l)	1.02 ± 0.33	1.06 ± 0.35	1.02 ± 0.32	**<0.001**	1.07 ± 0.38	1.02 ± 0.33	**<0.001**	1.06 ± 0.35	1.02 ± 0.32	**<0.001**
Non-HDL-C (mmol/l)	4.69 ± 0.33	4.67 ± 1.04	4.70 ± 0.99	0.052	4.68 ± 1.03	4.69 ± 1.00	0.802	4.66 ± 1.04	4.70 ± 0.99	**0.012**
TG/HDL-C ratio	2.17 ± 0.92	2.36 ± 1.00	2.14 ± 0.90	**<0.001**	2.37 ± 0.98	2.17 ± 0.92	**<0.001**	2.36 ± 1.00	2.13 ± 0.90	**<0.001**

Bold indicates that the *P* value is less than 0.05.

### Associations between maternal lipid profiles and lipid-derived indicators in early pregnancy and the risk of GDM

**Fig. 1:**
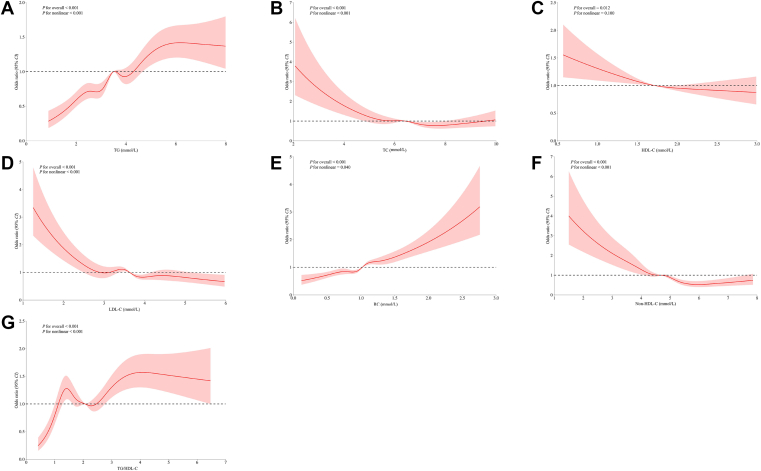
Restricted cubic spline-based modeling for the associations between maternal lipid profiles and lipid-derived indicators in the early pregnancy and GDM. (A) TG; (B) TC; (C) HDL-C; (D) LDL-C; (E) RC; (F) Non-HDL-C; (G) TG/HDL-C ratio. The model was adjusted for maternal age, residence location, education, ethnicity, monthly household income, maternal prepregnancy BMI, drinking before pregnancy, smoking before pregnancy, drinking in early pregnancy, smoking in early pregnancy, parity, family history of diabetes, family history of hyperglycemia, and other lipid indicators (TG, TC, LDL-C, HDL-C, RC, TG/HDL-C ratio, and non-HDL-C), except for the grouping variables.

The associations between maternal lipid profiles and lipid-derived indicators and the risk of GDM determined by logistic regression models are shown in Table 2. After adjusting for potential confounders, significant positive associations were observed between TG, RC, and TG/HDL-C ratio and the risk of GDM, whereas TC, HDL-C, LDL-C, and non-HDL-C showed significant negative associations. However, a significant increase in the risk of GDM was observed with increasing quartiles of TG, TC, and RC compared with the lowest quartile (Q1) after adjusting for potential confounders. For TG, the OR and 95% CI for Q3 and Q4 were 1.456 (95% CI: 1.245, 1.704) and 1.646 (95% CI: 1.363, 1.988), respectively. For TC, the OR and 95% CI for Q3 and Q4 were 1.366 (95% CI: 1.096, 1.704) and 1.654 (1.241, 2.205), respectively. For RC, the OR and 95% CI for Q3 and Q4 were 1.281 (95% CI: 1.122, 1.462) and 1.396 (95% CI: 1.189, 1.640), respectively. In contrast, a significant reduction in the risk of GDM was observed with increasing quartiles of non-HDL-C and TG/HDL-C ratio. For non-HDL-C, the OR and 95% CI for Q2, Q3, and Q4 were 0.741 (95% CI: 0.628, 0.874), 0.640 (95% CI: 0.502, 0.816), and 0.412 (95% CI: 0.301, 0.564), respectively. For TG/HDL-C ratio, the OR and 95% CI for Q3 was 0.813 (95% CI: 0.686, 0.965). After the quadruple grouping, there were significant linear associations between TG, TC, RC, and non-HDL-C and the risk of GDM (*P*_trend_ <0.05). In the multivariable-adjusted RCS-based model, the nonlinear associations between TG, TC, LDL-C, RC, non-HDL-C, and TG/HDL-C ratio and GDM were significant (*P*_nonlinear_ <0.05, Fig. 1), whereas evidence of nonlinear associations for HDL-C was lacking.

**Table 2:** Associations between maternal lipid profiles and lipid-derived indicators in early pregnancy and the risk of GDM

Variables	Model 1^a^		Model 2^b^		Model 3^c^	
	OR (95% CI)	*P*	OR (95% CI)	*P*	OR (95% CI)	*P*
TG (mmol/l)	1.216 (1.188–1.245)	**<0.001**	1.187 (1.159–1.216)	**<0.001**	1.171 (1.116–1.229)	**<0.001**
TG quartile						
Q1 (<2.76)	Reference (1.000)		Reference (1.000)		Reference (1.000)	
Q2 (2.76–3.50)	1.110 (1.011–1.218)	**0.028**	1.058 (0.963–1.163)	0.242	1.107 (0.982–1.248)	0.098
Q3 (3.50–4.22)	1.423 (1.301–1.556)	**<0.001**	1.379 (1.260–1.510)	**<0.001**	1.456 (1.245–1.704)	**<0.001**
Q4 (≥4.22)	1.825 (1.674–1.990)	**<0.001**	1.685 (1.543–1.840)	**<0.001**	1.646 (1.363–1.988)	**<0.001**
*P*_trend_	**<0.001**		**<0.001**		**<0.001**	
TC (mmol/l)	0.959 (0.934–0.985)	**0.002**	0.991 (0.965–1.018)	0.517	0.898 (0.824–0.979)	**0.015**
TC quartile						
Q1 (<5.68)	Reference (1.000)		Reference (1.000)		Reference (1.000)	
Q2 (5.68–6.41)	0.785 (0.717–0.859)	**<0.001**	0.835 (0.762–0.916)	**<0.001**	1.039 (0.890–1.214)	0.626
Q3 (6.41–7.14)	0.944 (0.870–1.023)	0.944	1.029 (0.948–1.118)	0.494	1.366 (1.096–1.704)	**0.006**
Q4 (≥7.14)	0.895 (0.823–0.974)	**0.010**	0.998 (0.915–1.088)	0.961	1.654 (1.241–2.205)	**0.001**
*P*_trend_	0.192		0.205		**0.001**	
HDL-C (mmol/l)	0.796 (0.726–0.874)	**<0.001**	0.851 (0.773–0.936)	**0.001**	0.802 (0.672–0.957)	**0.014**
HDL-C quartile						
Q1 (<1.54)	Reference (1.000)		Reference (1.000)		Reference (1.000)	
Q2 (1.54–1.72)	0.859 (0.785–0.940)	**0.001**	0.875 (0.798–0.958)	**0.004**	0.917 (0.828–1.016)	0.098
Q3 (1.72–1.94)	0.886 (0.817–0.960)	**0.003**	0.921 (0.849–1.000)	0.051	0.932 (0.833–1.044)	0.225
Q4 (≥1.94)	0.835 (0.767–0.909)	**<0.001**	0.886 (0.812–0.967)	**0.007**	0.918 (0.793–1.062)	0.250
*P*_trend_	**<0.001**		**0.022**		0.313	
LDL-C (mmol/l)	0.882 (0.847–0.918)	**<0.001**	0.931 (0.893–0.970)	**0.001**	0.741 (0.667–0.822)	**<0.001**
LDL-C quartile						
Q1 (<3.15)	Reference (1.000)		Reference (1.000)		Reference (1.000)	
Q2 (3.15–3.63)	0.787 (0.719–0.862)	**<0.001**	0.822 (0.749–0.901)	**<0.001**	0.902 (0.772–1.054)	0.196
Q3 (3.63–4.14)	0.910 (0.840–0.985)	**0.020**	0.978 (0.902–1.061)	0.600	0.978 (0.791–1.210)	0.839
Q4 (≥4.14)	0.812 (0.746–0.884)	**<0.001**	0.908 (0.832–0.991)	**0.030**	0.991 (0.758–1.295)	0.947
*P*_trend_	**<0.001**		0.323		0.962	
RC (mmol/l)	1.466 (1.340–1.604)	**<0.001**	1.507 (1.376–1.651)	**<0.001**	1.915 (1.614–2.272)	**<0.001**
RC quartile						
Q1 (<0.81)	Reference (1.000)		Reference (1.000)		Reference (1.000)	
Q2 (0.81–1.02)	1.006 (0.920–1.100)	0.898	1.056 (0.965–1.157)	0.237	1.103 (0.992–1.226)	0.071
Q3 (1.02–1.19)	1.160 (1.064–1.265)	**0.001**	1.210 (1.108–1.321)	**<0.001**	1.281 (1.122–1.462)	**<0.001**
Q4 (≥1.19)	1.270 (1.167–1.382)	**<0.001**	1.313 (1.205–1.432)	**<0.001**	1.396 (1.189–1.640)	**<0.001**
*P*_trend_	**<0.001**		**<0.001**		**<0.001**	
Non-HDL-C (mmol/l)	0.971 (0.942–1.000)	0.052	1.005 (0.975–1.037)	0.735	0.833 (0.758–0.916)	**<0.001**
Non-HDL-C quartile						
Q1 (<4.02)	Reference (1.000)		Reference (1.000)		Reference (1.000)	
Q2 (4.02–4.69)	0.837 (0.766–0.914)	**<0.001**	0.864 (0.790–0.945)	**0.001**	0.741 (0.628–0.874)	**<0.001**
Q3 (4.69–5.30)	0.979 (0.902–1.062)	0.603	1.057 (0.972–1.149)	0.197	0.640 (0.502–0.816)	**<0.001**
Q4 (≥5.30)	0.876 (0.804–0.954)	**0.002**	0.952 (0.872–1.038)	0.267	0.412 (0.301–0.564)	**<0.001**
*P*_trend_	0.070		0.736		**<0.001**	
TG/HDL-C ratio	1.273 (1.235–1.312)	**<0.001**	1.230 (1.193–1.269)	**<0.001**	1.164 (1.087–1.247)	**<0.001**
TG/HDL-C ratio quartile						
Q1 (<1.53)	Reference (1.000)		Reference (1.000)		Reference (1.000)	
Q2 (1.53–2.07)	1.147 (1.047–1.256)	**0.003**	1.109 (1.011–1.216)	**0.028**	0.969 (0.853–1.100)	0.625
Q3 (2.07–2.57)	1.239 (1.132–1.355)	**<0.001**	1.183 (1.080–1.296)	**<0.001**	0.813 (0.686–0.965)	**0.018**
Q4 (≥2.57)	1.784 (1.638–1.943)	**<0.001**	1.620 (1.485–1.767)	**<0.001**	0.992 (0.801–1.229)	0.941
*P*_trend_	**<0.001**		**<0.001**		0.738	

Bold indicates that the *P* value is less than 0.05.

a Model 1 was unadjusted.

b Model 2 was adjusted for maternal age, residence location, education, ethnicity, monthly household income, maternal prepregnancy BMI, drinking before pregnancy, smoking before pregnancy, drinking in early pregnancy, smoking in early pregnancy, parity, family history of diabetes, and family history of hyperglycemia.

c Model 3 was further adjusted for other lipid profiles and lipid-derived indicators (TG, TC, LDL-C, HDL-C, RC, TG/HDL-C ratio, and non-HDL-C), except for the grouping variables based on model 2.

### Associations between maternal lipid profiles and lipid-derived indicators in early pregnancy and the risk of PE

**Fig. 2:**
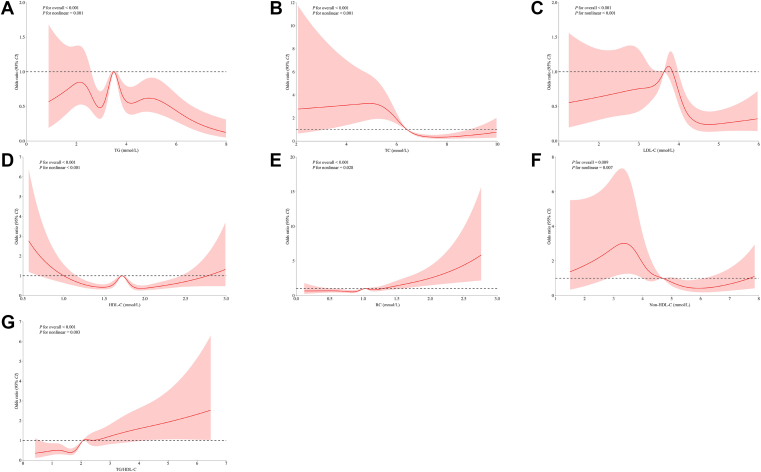
Restricted cubic spline-based modeling for the associations between maternal lipid profiles and lipid-derived indicators in the early pregnancy and PE. (A) TG; (B) TC; (C) LDL-C; (D) HDL-C; (E) RC; (F) Non-HDL-C; (G) TG/HDL-C ratio. The model was adjusted for maternal age, residence location, education, ethnicity, monthly household income, maternal prepregnancy BMI, drinking before pregnancy, smoking before pregnancy, drinking in early pregnancy, smoking in early pregnancy, parity, family history of diabetes, family history of hyperglycemia, and other lipid indicators (TG, TC, LDL-C, HDL-C, RC, TG/HDL-C ratio, and non-HDL-C), except for the grouping variables.

The associations between maternal lipid profiles and lipid-derived indicators and the risk of PE determined by logistic regression models are shown in Table 3. After adjusting for potential confounders, significant positive associations were observed between RC and TG/HDL-C ratio and the risk of PE, whereas TG showed significant negative associations. However, a significant increase in the risk of PE was observed with increasing quartiles of HDL-C and TG/HDL-C ratio compared with the lowest quartile (Q1) after adjusting for potential confounders. For HDL-C, the OR and 95% CI for Q2 and Q3 were 1.706 (95% CI: 1.301, 2.238) and 1.598 (95% CI: 1.170, 2.183), respectively. For TG/HDL-C ratio, the OR and 95% CI for Q3 and Q4 were 1.935 (95% CI: 1.216, 3.078) and 2.451 (95% CI: 1.369, 4.388), respectively. In contrast, a significant reduction in the risk of PE was observed with increasing quartiles of TG. For TG, the OR and 95% CI for Q4 was 0.567 (95% CI: 0.334, 0.962). After the quartile grouping, there were significant linear associations between TG and TG/HDL-C ratio and the risk of PE (*P*_trend_ <0.05). In the multivariable-adjusted RCS-based model, the nonlinear associations between TG, TC, HDL-C, LDL-C, RC, non-HDL-C, and TG/HDL-C ratio and PE were significant (*P*_nonlinear_ <0.05, Fig. 2).

**Table 3:** Associations between maternal lipid profiles and lipid-derived indicators in early pregnancy and the risk of PE

Variables	Model 1^a^		Model 2^b^		Model 3^c^	
	OR (95% CI)	*P*	OR (95% CI)	*P*	OR (95% CI)	*P*
TG (mmol/l)	1.117 (1.049–1.188)	**0.001**	1.089 (1.023–1.161)	**0.008**	0.792 (0.688–0.912)	**0.001**
TG quartile						
Q1 (<2.76)	Reference (1.000)		Reference (1.000)		Reference (1.000)	
Q2 (2.76–3.50)	1.098 (0.847–1.422)	0.481	1.039 (0.799–1.351)	0.773	0.847 (0.602–1.193)	0.342
Q3 (3.50–4.22)	1.820 (1.441–2.299)	**<0.001**	1.840 (1.451–2.333)	**<0.001**	0.988 (0.642–1.521)	0.957
Q4 (≥4.22)	1.488 (1.168–1.895)	**0.001**	1.381 (1.079–1.769)	**0.010**	0.567 (0.334–0.962)	**0.035**
*P*_trend_	**<0.001**		**<0.001**		**0.042**	
TC (mmol/l)	0.945 (0.881–1.015)	0.119	1.064 (0.990–1.143)	0.091	1.021 (0.808–1.290)	0.863
TC quartile						
Q1 (<5.68)	Reference (1.000)		Reference (1.000)		Reference (1.000)	
Q2 (5.68–6.41)	0.939 (0.738–1.195)	0.612	1.176 (0.919–1.504)	0.198	1.285 (0.829–1.992)	0.262
Q3 (6.41–7.14)	1.082 (0.872–1.342)	0.474	1.452 (1.163–1.813)	**0.001**	1.140 (0.615–2.111)	0.678
Q4 (≥7.14)	0.896 (0.711–1.131)	0.356	1.300 (1.022–1.654)	**0.032**	1.438 (0.650–3.179)	0.370
*P*_trend_	0.650		**0.006**		0.465	
HDL-C (mmol/l)	0.545 (0.423–0.703)	**<0.001**	0.788 (0.609–1.020)	0.070	0.796 (0.490–1.294)	0.358
HDL-C quartile						
Q1 (<1.54)	Reference (1.000)		Reference (1.000)		Reference (1.000)	
Q2 (1.54–1.72)	1.264 (1.004–1.593)	**0.047**	1.485 (1.173–1.880)	**0.001**	1.706 (1.301–2.238)	**<0.001**
Q3 (1.72–1.94)	1.157 (0.933–1.435)	0.183	1.467 (1.177–1.829)	**0.001**	1.598 (1.170–2.183)	**0.003**
Q4 (≥1.94)	0.696 (0.540–0.898)	**0.005**	0.963 (0.742–1.248)	0.774	1.415 (0.934–2.144)	0.101
*P*_trend_	**0.011**		0.769		0.114	
LDL-C (mmol/l)	0.900 (0.809–1.002)	0.055	1.060 (0.951–1.181)	0.292	0.777 (0.588–1.027)	0.076
LDL-C quartile						
Q1 (<3.15)	Reference (1.000)		Reference (1.000)		Reference (1.000)	
Q2 (3.15–3.63)	0.812 (0.630–1.046)	0.108	0.942 (0.728–1.219)	0.650	0.986 (0.644–1.510)	0.948
Q3 (3.63–4.14)	1.250 (1.016–1.536)	**0.035**	1.591 (1.286–1.968)	**<0.001**	1.729 (0.974–3.068)	0.061
Q4 (≥4.14)	0.759 (0.596–0.967)	**0.026**	1.064 (0.829–1.366)	0.626	0.791 (0.389–1.609)	0.517
*P*_trend_	0.455		**0.027**		0.656	
RC (mmol/l)	1.555 (1.230–1.965)	**<0.001**	1.848 (1.466–2.331)	**<0.001**	2.548 (1.618–4.014)	**<0.001**
RC quartile						
Q1 (<0.81)	Reference (1.000)		Reference (1.000)		Reference (1.000)	
Q2 (0.81–1.02)	0.811 (0.633–1.040)	0.098	0.938 (0.729–1.207)	0.619	0.897 (0.668–1.206)	0.473
Q3 (1.02–1.19)	1.157 (0.925–1.449)	0.202	1.365 (1.086–1.717)	**0.008**	1.070 (0.735–1.558)	0.724
Q4 (≥1.19)	1.191 (0.954–1.488)	0.122	1.455 (1.159–1.826)	**0.001**	1.386 (0.884–2.172)	0.155
*P*_trend_	**0.017**		**<0.001**		0.137	
Non-HDL-C (mmol/l)	0.990 (0.913–1.073)	0.802	1.109 (1.023–1.203)	**0.012**	1.117 (0.867–1.439)	0.394
Non-HDL-C quartile						
Q1 (<4.02)	Reference (1.000)		Reference (1.000)		Reference (1.000)	
Q2 (4.02–4.69)	0.877 (0.690–1.115)	0.285	1.012 (0.792–1.292)	0.925	0.704 (0.442–1.124)	0.141
Q3 (4.69–5.30)	1.082 (0.870–1.346)	0.479	1.352 (1.082–1.691)	**0.008**	0.501 (0.251–0.999)	0.050
Q4 (≥5.30)	0.955 (0.760–1.200)	0.693	1.303 (1.029–1.650)	**0.028**	0.700 (0.297–1.653)	0.416
*P*_trend_	0.849		**0.004**		0.445	
TG/HDL-C ratio	1.235 (1.143–1.335)	**<0.001**	1.149 (1.060–1.246)	**0.001**	1.300 (1.084–1.559)	**0.005**
TG/HDL-C ratio quartile						
Q1 (<1.53)	Reference (1.000)		Reference (1.000)		Reference (1.000)	
Q2 (1.53–2.07)	1.145 (0.875–1.499)	0.324	1.020 (0.777–1.339)	0.885	1.126 (0.781–1.625)	0.525
Q3 (2.07–2.57)	1.978 (1.553–2.521)	**<0.001**	1.804 (1.412–2.306)	**<0.001**	1.935 (1.216–3.078)	**0.005**
Q4 (≥2.57)	1.915 (1.502–2.442)	**<0.001**	1.535 (1.198–1.967)	**0.001**	2.451 (1.369–4.388)	**0.003**
*P*_trend_	**<0.001**		**<0.001**		**<0.001**	

Bold indicates that the *P* value is less than 0.05.

a Model 1 was unadjusted.

b Model 2 was adjusted for maternal age, residence location, education, ethnicity, monthly household income, maternal prepregnancy BMI, drinking before pregnancy, smoking before pregnancy, drinking in early pregnancy, smoking in early pregnancy, parity, family history of diabetes, and family history of hyperglycemia.

c Model 3 was further adjusted for other lipid profiles and lipid-derived indicators (TG, TC, LDL-C, HDL-C, RC, TG/HDLC ratio, and non-HDL-C), except for the grouping variables based on model 2.

### Associations between maternal lipid profiles and lipid-derived indicators in early pregnancy and the risk of GDM/PE

**Fig. 3:**
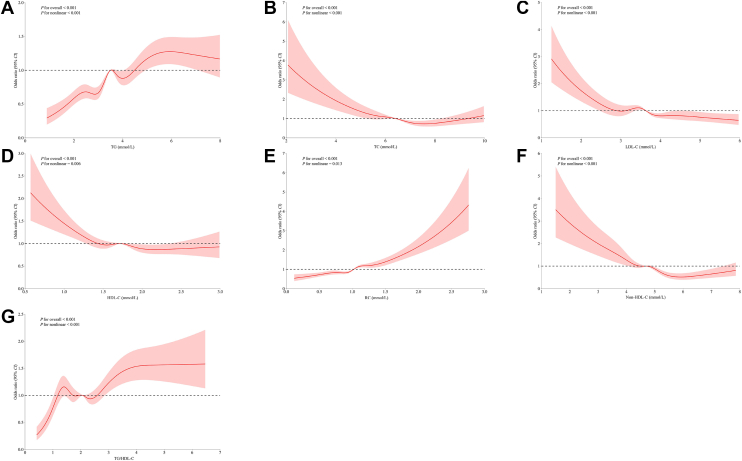
Restricted cubic spline-based modeling for the associations between maternal lipid profiles and lipid-derived indicators in the early pregnancy and GDM/PE. (A) TG; (B) TC; (C) LDL-C; (D) HDL-C; (E) RC; (F) Non-HDL-C; (G) TG/HDL-C ratio. The model was adjusted for maternal age, residence location, education, ethnicity, monthly household income, maternal prepregnancy BMI, drinking before pregnancy, smoking before pregnancy, drinking in early pregnancy, smoking in early pregnancy, parity, family history of diabetes, family history of hyperglycemia, and other lipid indicators (TG, TC, LDL-C, HDL-C, RC, TG/HDL-C ratio, and non-HDL-C), except for the grouping variables.

The associations between maternal lipid profiles and lipid-derived indicators and the risk of GDM/PE determined by logistic regression models are shown in Table 4. After adjusting for potential confounders, significant positive associations were observed between TG, RC, and TG/HDL-C ratio and the risk of GDM/PE, whereas HDL-C, LDL-C, and non-HDL-C showed significant negative associations. However, a significant increase in the risk of GDM/PE was observed with increasing quartiles of TG, TC, and RC compared with the lowest quartile (Q1) after adjusting for potential confounders. For TG, the OR and 95% CI for Q3 and Q4 were 1.479 (95% CI: 1.272, 1.721) and 1.564 (95% CI: 1.302, 1.877), respectively. For TC, the OR and 95% CI for Q3 and Q4 were 1.321 (95% CI: 1.067, 1.636) and 1.655 (95% CI: 1.253, 2.186), respectively. For RC, the OR and 95% CI for Q3 and Q4 were 1.233 (95% CI: 1.085, 1.401) and 1.379 (95% CI: 1.180, 1.612), respectively. In contrast, a significant reduction in the risk of GDM/PE was observed with increasing quartiles of non-HDL-C. For non-HDL-C, the OR and 95% CI for Q2, Q3, and Q4 were 0.741 (95% CI: 0.632, 0.870), 0.658 (95% CI: 0.520, 0.832), and 0.424 (95% CI: 0.313, 0.575), respectively. After the quartile grouping, there were significant linear associations between TG, TC, RC, and non-HDL-C and the risk of GDM/PE (*P*_trend_ <0.05). In the multivariable-adjusted RCS-based model, the nonlinear associations between TG, TC, HDL-C, LDL-C, RC, and non-HDL-C and GDM/PE were significant (*P*_nonlinear_ <0.05, Fig. 3).

**Table 4:** Associations between maternal lipid profiles and lipid-derived indicators in early pregnancy and the risk of GDM/PE

Variables	Model 1^a^		Model 2^b^		Model 3^c^	
	OR (95% CI)	*P*	OR (95% CI)	*P*	OR (95% CI)	*P*
TG (mmol/l)	1.209 (1.182–1.237)	**<0.001**	1.180 (1.153–1.208)	**<0.001**	1.144 (1.091–1.199)	**<0.001**
TG quartile						
Q1 (<2.76)	Reference (1.000)		Reference (1.000)		Reference (1.000)	
Q2 (2.76–3.50)	1.108 (1.013–1.211)	**0.025**	1.054 (0.963–1.155)	0.254	1.097 (0.976–1.232)	0.120
Q3 (3.50–4.22)	1.477 (1.356–1.609)	**<0.001**	1.440 (1.320–1.570)	**<0.001**	1.479 (1.272–1.721)	**<0.001**
Q4 (≥4.22)	1.797 (1.653–1.953)	**<0.001**	1.662 (1.526–1.809)	**<0.001**	1.564 (1.302–1.877)	**<0.001**
*P*_trend_	**<0.001**		**<0.001**		**<0.001**	
TC (mmol/l)	0.949 (0.925–0.974)	**<0.001**	0.991 (0.966–1.018)	0.509	0.926 (0.852–1.007)	0.072
TC quartile						
Q1 (<5.68)	Reference (1.000)		Reference (1.000)		Reference (1.000)	
Q2 (5.68–6.41)	0.797 (0.731–0.870)	**<0.001**	0.864 (0.790–0.944)	**0.001**	1.071 (0.922–1.244)	0.369
Q3 (6.41–7.14)	0.925 (0.855–0.999)	**0.049**	1.033 (0.954–1.119)	0.427	1.321 (1.067–1.636)	**0.011**
Q4 (≥7.14)	0.871 (0.803–0.945)	**0.001**	1.002 (0.921–1.089)	0.971	1.655 (1.253–2.186)	**<0.001**
*P*_trend_	**0.026**		0.225		**<0.001**	
HDL-C (mmol/l)	0.748 (0.683–0.818)	**<0.001**	0.829 (0.756–0.909)	**<0.001**	0.774 (0.652–0.917)	**0.003**
HDL-C quartile						
Q1 (<1.54)	Reference (1.000)		Reference (1.000)		Reference (1.000)	
Q2 (1.54–1.72)	0.890 (0.816–0.970)	**0.008**	0.921 (0.843–1.006)	0.066	0.971 (0.88–1.072)	0.564
Q3 (1.72–1.94)	0.902 (0.834–0.974)	**0.009**	0.960 (0.887–1.040)	0.317	0.975 (0.874–1.087)	0.645
Q4 (≥1.94)	0.803 (0.739–0.872)	**<0.001**	0.880 (0.809–0.958)	**0.003**	0.929 (0.806–1.071)	0.309
*P*_trend_	**<0.001**		**0.013**		0.380	
LDL-C (mmol/l)	0.871 (0.838–0.905)	**<0.001**	0.932 (0.896–0.970)	**<0.001**	0.752 (0.680–0.832)	**<0.001**
LDL-C quartile						
Q1 (<3.15)	Reference (1.000)		Reference (1.000)		Reference (1.000)	
Q2 (3.15–3.63)	0.789 (0.723–0.861)	**<0.001**	0.832 (0.761–0.909)	**<0.001**	0.904 (0.778–1.050)	0.187
Q3 (3.63–4.14)	0.905 (0.838–0.977)	**0.010**	0.992 (0.917–1.073)	0.835	0.981 (0.800–1.204)	0.855
Q4 (≥4.14)	0.788 (0.726–0.855)	**<0.001**	0.905 (0.832–0.985)	**0.021**	0.969 (0.748–1.256)	0.814
*P*_trend_	**<0.001**		0.297		0.910	
RC (mmol/l)	1.464 (1.343–1.597)	**<0.001**	1.538 (1.409–1.680)	**<0.001**	2.085 (1.766–2.461)	**<0.001**
RC quartile						
Q1 (<0.81)	Reference (1.000)		Reference (1.000)		Reference (1.000)	
Q2 (0.81–1.02)	0.978 (0.898–1.066)	0.614	1.041 (0.954–1.136)	0.364	1.079 (0.974–1.196)	0.145
Q3 (1.02–1.19)	1.136 (1.046–1.234)	**0.003**	1.203 (1.106–1.309)	**<0.001**	1.233 (1.085–1.401)	**0.001**
Q4 (≥1.19)	1.236 (1.139–1.34)	**<0.001**	1.305 (1.201–1.418)	**<0.001**	1.379 (1.180–1.612)	**<0.001**
*P*_trend_	**<0.001**		**<0.001**		**<0.001**	
Non-HDL-C (mmol/l)	0.963 (0.936–0.992)	**0.012**	1.008 (0.979–1.039)	0.589	0.871 (0.795–0.954)	**0.003**
Non-HDL-C quartile						
Q1 (<4.02)	Reference (1.000)		Reference (1.000)		Reference (1.000)	
Q2 (4.02–4.69)	0.829 (0.762–0.902)	**<0.001**	0.864 (0.793–0.942)	**0.001**	0.741 (0.632–0.870)	**<0.001**
Q3 (4.69–5.30)	0.970 (0.896–1.049)	0.446	1.066 (0.984–1.156)	0.120	0.658 (0.520–0.832)	**<0.001**
Q4 (≥5.30)	0.852 (0.785–0.925)	**<0.001**	0.950 (0.873–1.033)	0.230	0.424 (0.313–0.575)	**<0.001**
*P*_trend_	**0.012**		0.708		**<0.001**	
TG/HDL-C ratio	1.281 (1.244–1.319)	**<0.001**	1.232 (1.195–1.269)	**<0.001**	1.196 (1.119–1.278)	**<0.001**
TG/HDL-C ratio quartile						
Q1 (<1.53)	Reference (1.000)		Reference (1.000)		Reference (1.000)	
Q2 (1.53–2.07)	1.154 (1.057–1.260)	**0.001**	1.107 (1.013–1.211)	**0.025**	0.967 (0.855–1.094)	0.594
Q3 (2.07–2.57)	1.306 (1.198–1.424)	**<0.001**	1.241 (1.137–1.354)	**<0.001**	0.859 (0.729–1.012)	0.070
Q4 (≥2.57)	1.811 (1.667–1.966)	**<0.001**	1.620 (1.489–1.763)	**<0.001**	1.035 (0.842–1.272)	0.747
*P*_trend_	**<0.001**		**<0.001**		0.847	

Bold indicates that the *P* value is less than 0.05.

a Model 1 was unadjusted.

b Model 2 was adjusted for maternal age, residence location, education, ethnicity, monthly household income, maternal prepregnancy BMI, drinking before pregnancy, smoking before pregnancy, drinking in early pregnancy, smoking in early pregnancy, parity, family history of diabetes, and family history of hyperglycemia.

c Model 3 was further adjusted for other lipid profiles and lipid-derived indicators (TG, TC, LDL-C, HDL-C, RC, TG/HDLC ratio, and non-HDL-C), except for the grouping variables based on model 2.

### Subgroup analysis

To further evaluate the robustness of the results, this study investigated the potential interaction effects of each covariate with lipid profiles or lipid-derived indicators significantly associated with the outcome in the corresponding outcome risk analyses based on model 3. Significant interaction effects were observed between maternal age and RC, TG, TC, and non-HDL-C in relation to GDM risk, whereas interactions with TG and TG/HDL-C ratio were significant for PE risk. For GDM/PE risk, maternal age showed significant interactions with RC, TG, and TC. Residence location demonstrated significant interaction effects with RC for both GDM and GDM/PE risk. Educational level exhibited significant interactions with RC, non-HDL-C, and TG/HDL-C ratio for GDM risk, with TG, HDL-C, and TG/HDL-C ratio for PE risk, and with RC for GDM/PE risk. Prepregnancy maternal BMI showed significant interactions with RC, TG, TC, and TG/HDL-C ratio for GDM risk, with TG for PE risk, and with RC, TG, TC, and non-HDL-C for GDM/PE risk. Parity demonstrated significant interactions with TG, TC, non-HDL-C, and TG/HDL-C ratio for GDM risk, with TG and TG/HDL-C ratio for PE risk, and with TG, TC, and non-HDL-C for GDM/PE risk. Family history of hyperglycemia showed a significant interaction effect only with HDL-C for PE risk. Subgroup analyses stratified by these covariates with significant interactions demonstrated that the association between lipid profiles or lipid-derived indicators and the risks of GDM, PE, and GDM/PE remained largely consistent across subgroups, indicating stable and reliable findings. The detailed results are presented in Supplemental Tables S2–S7.

### Mediation analysis

**Fig. 4:**
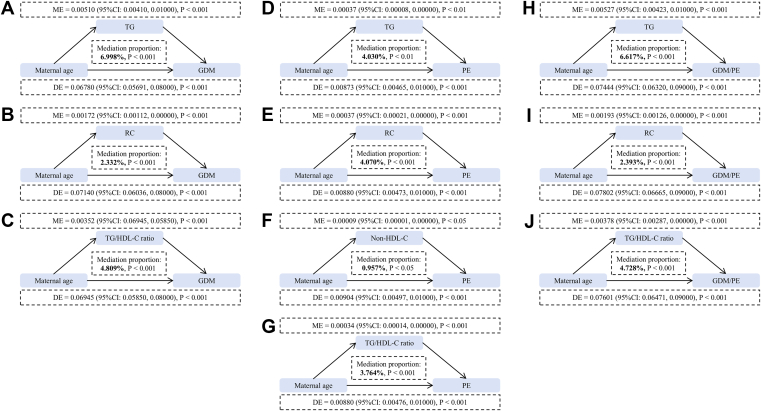
Mediation analyses of maternal lipid profiles and lipid-derived indicators in the early pregnancy in the associations between maternal age and outcomes. A–C: Mediation analysis for GDM. D–G: Mediation analysis for PE. H–J: Mediation analysis for GDM/PE. Adjusted for maternal age, residence location, education, ethnicity, monthly household income, maternal prepregnancy BMI, drinking before pregnancy, smoking before pregnancy, drinking in early pregnancy, smoking in early pregnancy, parity, family history of diabetes, family history of hyperglycemia, and other lipid profiles and lipid-derived indicators (TG, TC, LDL-C, HDL-C, RC, TG/HDL-C ratio, and non-HDL-C), except for the mediation variables.

As shown in Fig. 4 and Supplemental Table S8, maternal early pregnancy TG, RC, and TG/HDL-C ratio partially mediated the association between maternal age and the development of GDM, PE, and GDM/PE during pregnancy. In addition, non-HDL-C partially mediated the association between maternal age and the development of PE. Specifically, in the association between maternal age and GDM, the proportion of mediating effect of TG, RC, and TG/HDL-C ratio was 6.998%, 2.332%, and 4.809%, respectively. In the association between maternal age and PE, the proportion of mediating effect of TG, RC, non-HDL-C, and TG/HDL-C ratio was 4.030%, 4.070%, 0.957%, and 3.764%, respectively. In the association between maternal age and GDM/PE, the proportion of mediating effect of TG, RC, and TG/HDL-C ratio was 6.617%, 2.393%, and 4.728%, respectively.

## Discussion

This prospective cohort study included four basic lipid profiles and three lipid-derived indicators to systematically assess their associations with GDM, PE, and GDM/PE. The main findings are as follows: *1*) TG, TC, and RC in the first trimester were significantly positively associated with GDM and GDM/PE risk. The TG/HDL-C ratio was positively associated with PE risk. Notably, non-HDL-C was negatively associated with GDM and GDM/PE risks, and HDL-C was positively associated with PE risk. Subgroup analyses confirmed the stability of the results. *2*) The RCS curve showed that TG, TC, LDL-C, RC, non-HDL-C, and the TG/HDL-C ratio all had a nonlinear dose-response relationship with the above three outcomes. HDL-C had a nonlinear association only with PE and GDM/PE. *3*) Maternal TG, RC, and TG/HDL-C ratio in the first trimester partially mediated the associations between maternal age and the risks of GDM, PE, and GDM/PE. Non-HDL-C also partially mediated the association between maternal age and the PE risk.

The results of this study indicate a highly consistent pattern of statistically significant positive or negative associations between lipid markers and GDM and GDM/PE risks. Consistent with previous studies, this study found that TG and TC were positively associated with the GDM and GDM/PE risk (44, 45). RC was also found to be an independent risk factor for GDM and GDM/PE in this study, which was consistent with the results of the Chinese and Korean cohort studies (29, 30, 31). Notably, this study revealed negative associations between non-HDL-C and GDM and GDM/PE risks, contrary to common sense. However, this paradoxical observation was not isolated. Cibickova *et al*. (34) found that there was no significant difference in non-HDL-C levels between pregnant women with and without GDM. Two other studies reported that non-HDL-C was inversely associated with mortality in patients with coronary artery disease and in hemodialysis patients, respectively (46, 47). Non-HDL-C is generally considered to be the sum of atherogenic lipids (including LDL-C, VLDL-C, and IDL-C.), and its elevated level is theoretically associated with worse outcomes. Wang *et al*. (46) suggested that this paradoxical association in the hemodialysis population may be related to malnutrition. Since this study only collected BMI data and did not obtain more detailed nutritional information, such as dietary intake, the confounding effect of nutritional status cannot be ruled out. There may also be other unidentified confounding factors (such as metabolic compensation mechanisms during pregnancy) that may have influenced this association.

The role of lipids in the pathogenesis of GDM has not been fully clarified. Previous studies have associated dyslipidemia with glucose metabolism disorders, which play a key role in the pathogenesis of diabetes. It is reported that excess cholesterol may disrupt pancreatic β-cell insulin homeostasis (48). Furthermore, lipotoxicity can also cause cell death via apoptosis (49). IR and inflammation may also account for the association between lipids and GDM (29). A Chinese study reported that RC was associated with diabetes, prediabetes, and IR (50). Inflammatory markers may partially mediate these associations (51, 52). Increased inflammatory cytokines can impair insulin secretion and sensitivity and induce IR and β-cell dysfunction (53). In addition, increasing evidence suggests that elevated TG is strongly associated with an increased risk of GDM (17, 44). TG was consistently associated with insulin index and glucose metabolism index in both the first and second trimesters (54).

Existing research conclusions regarding the associations between blood lipids and PE risk are not entirely consistent. Some studies have shown that abnormal blood lipid levels before and during pregnancy are associated with the PE risk (45, 55). However, Chalas *et al*. (56) found that concentrations of TG, TC, LDL-C, and other indicators did not differ significantly between PE women and pregnant controls. Emet *et al*. (57) also failed to find an association between lipid profile changes and PE risk. This study found that the TG/HDL-C ratio in early pregnancy was positively associated with PE risk. Of note, a positive association between HDL-C and PE was also observed in this study—a finding contrary to the common perception that HDL-C is protective. However, some studies have reported that HDL-C levels in PE patients were significantly higher than that those in normal pregnant women (58, 59). Stefanović *et al*. (60) also observed a similar trend, though it did not reach statistical significance. Additionally, other clinical intervention evidence has cast doubt on the role of elevated HDL-C in cardiac protection (61, 62, 63). The core contradiction is that HDL-C concentration and function are not exactly matched. Riwanto and Landmesser (64) e mphasized that HDL particles are complex in composition, and their function is greatly influenced by compositional modifications. Navab *et al*. (65) proposed that under conditions of systemic inflammation, oxidants produced by the inflammatory response can damage key components of HDL, causing it to lose its anti-inflammatory and antioxidant capabilities and even transform into proinflammatory particles, thereby exacerbating vascular endothelial damage. However, the results of this study do not aim to negate the protective role of HDL but rather suggest that the unique dissociation between HDL-C concentration and function in populations with pregnancy complications warrants further in-depth exploration in the future.

The pathogenic role of lipids in PE may involve complex mechanisms. First, lipid accumulation in endothelial cells directly impairs vascular homeostasis. Elevated TG levels reduce vasodilator production (66) and eliminate the defense against thromboxane A_2_ (67). This imbalance impairs trophoblast invasion and spiral artery dilation, exacerbating the defect in placental spiral artery remodeling that characterizes PE (68, 69). Second, dyslipolytic disorders and IR are related to PE development. Lipoprotein lipase and hepatic lipase activities are abnormal in PE patients, leading to the accumulation of remnant lipoproteins (70, 71), and prolonging TG circulation time. IR exacerbates this defect (72, 73). Third, abnormal lipid alterations may promote oxidative stress. The coexistence of reduced antioxidant activity with hyperlipidemia and excessive lipid peroxidation results in an imbalance between pro-oxidants and antioxidants, leading to oxidative stress (74). The production of lipid peroxidation products can promote decidual vasculopathy in PE through a mechanism similar to atherogenesis (75). Fourth, lipids are associated with inflammation and immune dysregulation. High levels of tumor necrosis factor-alpha and other cytokines in patients with PE are associated with lipids (76). Supraphysiologic levels of cholesterol during pregnancy promote systemic inflammation, affecting placental vascularization (77).

Further RCS analysis showed that, except for HDL-C, which was not significantly nonlinearly associated with GDM, the other indicators (TG, TC, LDL-C, RC, non-HDL-C, and TG/HDL-C ratio) were all nonlinearly associated with the three outcomes. Findings were consistent from a large retrospective study that observed nonlinear relationships between lipid parameters and prediabetes risk (78). T he above results suggested that the impact of lipid metabolism on pregnancy complications was not a simple “linear cumulative effect” but rather involved dynamic regulation dependent on concentration intervals, reflecting the complexity and specificity of lipid mechanisms in different pregnancy outcomes. Furthermore, subgroup analyses demonstrated that the associations between various lipid parameters and different outcomes in this study remained largely consistent.

The mediating effect of lipid metabolism in the pathogenic pathway of high-risk factors for GDM or PE remains unknown. Due to factors, such as socioeconomic development, the three-child policy, longer education, and increased work pressure, the average childbearing age of Chinese women has risen (79), making advanced-age childbirth common. In view of this, the present study focused on early pregnancy lipids as the mediating variables in the association between maternal age and GDM, PE, and GDM/PE. The results indicate that TG, RC, and TG/HDL-C ratio during early pregnancy partially mediated the associations between maternal age and these three outcomes. Additionally, non-HDL-C also partially mediated the relationship between age and PE. In summary, these findings reveal the potential role of lipids in complications of advanced maternal age pregnancy and enhance our understanding of lipid biology during pregnancy. Despite the low mediation proportion, the statistical significance supports lipids as an intervenable metabolic pivot, providing a rationale for targeted management of advanced maternal age.

## Strengths and limitations

The data analysis in this study was conducted based on a prospective cohort with a larger sample size, minimizing recall bias and ensuring the study's reliability. However, some limitations remained. First, it should be noted that this is an observational study, and the results can only provide evidence of an association between lipids and GDM or PE. It cannot clarify whether there is a causal relationship between lipids and GDM or PE, or whether they are just markers of increased risk. Second, the participants in this study were all from a single hospital, which may have made the sample sources concentrated in a particular population, resulting in limited sample representativeness and affecting the extrapolation of the results. Third, we did not perform multistage lipid testing to reflect the association between lipid changes throughout pregnancy and GDM as well as PE. Finally, although we control for many confounding factors, the results may still be influenced by other potential confounding factors, such as information on lipid-lowering treatment before or during follow-up.

## Conclusion

This prospective cohort study evaluated associations between seven lipid parameters and GDM, PE, and GDM/PE, which found that first-trimester TG, TC, and RC were positively linked to GDM and GDM/PE, whereas TG/HDL-C ratio and HDL-C were associated with PE risk. However, non-HDL-C showed negative associations with GDM and GDM/PE. Nonlinear dose-response relationships were observed for most lipids with the outcomes. Subgroup analyses confirmed these associations were robust. In addition, early pregnancy TG, RC, and TG/HDL-C ratio partly mediated the effects of maternal age at pregnancy on all three outcomes. Non-HDL-C partly mediated the age-PE pathway. This study simultaneously included multiple lipid parameters for systematic analysis, revealing the impact of dyslipidemia on pregnancy outcomes from a more comprehensive perspective and providing richer evidence for exploring related mechanisms and clinical assessment.

## Ethical approval

This study was approved by the Ethics Committee of Xiangya School of Public Health, Central South University, and registered in the Chinese Clinical Trial Registry (registration number: ChiCTR1800016635; date of registration: June 14, 2018). All participants provided written informed consent prior to participating in our study, and this study complied with the ethical principles of the Helsinki Declaration of the World Medical Association.

## Data availability

The data underlying this article will be shared on reasonable request to the corresponding author.

## Supplemental data

This article contains supplemental data.

## Conflict of interest

The authors declare that they have no conflicts of interest with the contents of this article.

## References

[1] Wang H., Li N., Chivese T., Werfalli M., Sun H., Yuen L. (2022). IDF diabetes atlas: estimation of global and regional gestational diabetes mellitus prevalence for 2021 by international association of diabetes in pregnancy study group’s criteria. Diabetes Res. Clin. Pract..

[2] Gao C., Sun X., Lu L., Liu F., Yuan J. (2019). Prevalence of gestational diabetes mellitus in mainland China: a systematic review and meta-analysis. J. Diabetes Investig..

[3] Greco E., Calanducci M., Nicolaides K.H., Barry E.V.H., Huda M.S.B., Iliodromiti S. (2024). Gestational diabetes mellitus and adverse maternal and perinatal outcomes in twin and singleton pregnancies: a systematic review and meta-analysis. Am. J. Obstet. Gynecol..

[4] Ye W., Luo C., Huang J., Li C., Liu Z., Liu F. (2022). Gestational diabetes mellitus and adverse pregnancy outcomes: systematic review and meta-analysis. BMJ.

[5] Ackerman-Banks C.M., Palmsten K., Lipkind H.S., Ahrens K.A. (2024). Association between gestational diabetes and cardiovascular disease within 24 months postpartum. Am. J. Obstet. Gynecol. MFM.

[6] Daly B., Toulis K.A., Thomas N., Gokhale K., Martin J., Webber J. (2018). Increased risk of ischemic heart disease, hypertension, and type 2 diabetes in women with previous gestational diabetes mellitus, a target group in general practice for preventive interventions: a population-based cohort study. Plos Med..

[7] Magee L.A., Nicolaides K.H., Von Dadelszen P., Preeclampsia, Longo D.L. (2022). N. Engl. J. Med..

[8] Zhou S., Zhou N., Zhang H., Yang W., Liu Q., Zheng L. (2024). A prospective multicenter birth cohort in China: pregnancy health atlas. Eur. J. Epidemiol..

[9] Omani-Samani R., Ranjbaran M., Amini P., Esmailzadeh A., Sepidarkish M., Almasi-Hashiani A. (2019). Adverse maternal and neonatal outcomes in women with preeclampsia in Iran. J. Matern. Fetal. Neonatal. Med..

[10] Li X., Zhang W., Lin J., Liu H., Yang Z., Teng Y. (2021). Hypertensive disorders of pregnancy and risks of adverse pregnancy outcomes: a retrospective cohort study of 2368 patients. J. Hum. Hypertens..

[11] Wu P., Haththotuwa R., Kwok C.S., Babu A., Kotronias R.A., Rushton C. (2017). Preeclampsia and future cardiovascular health: a systematic review and meta-analysis. Circ. Cardiovasc. Qual. Outcomes.

[12] Pittara T., Vyrides A., Lamnisos D., Giannakou K. (2021). Pre-eclampsia and long-term health outcomes for mother and infant: an umbrella review. BJOG Int. J. Obstet. Gynaecol..

[13] Wang J., Yang W., Xiao W., Cao S. (2022). The association between smoking during pregnancy and hypertensive disorders of pregnancy: a systematic review and meta-analysis. Int. J. Gynecol. Obstet..

[14] Sun M., Luo M., Wang T., Wei J., Zhang S., Shu J. (2023). Effect of the interaction between advanced maternal age and pre-pregnancy BMI on pre-eclampsia and GDM in central China. BMJ Open Diabetes Res. Care.

[15] Zhang Y., Xiao C.M., Zhang Y., Chen Q., Zhang X.Q., Li X.F. (2021). Factors associated with gestational diabetes mellitus: a meta-analysis. J. Diabetes Res..

[16] Tesfa E., Nibret E., Munshea A. (2020). Maternal lipid profile and risk of pre-eclampsia in African pregnant women: a systematic review and meta-analysis. PLoS One.

[17] Hu J., Gillies C.L., Lin S., Stewart Z.A., Melford S.E., Abrams K.R. (2021). Association of maternal lipid profile and gestational diabetes mellitus: a systematic review and meta-analysis of 292 studies and 97,880 women. EClinicalMedicine.

[18] Wang C., Zhu W., Wei Y., Su R., Feng H., Hadar E. (2017). The associations between early pregnancy lipid profiles and pregnancy outcomes. J. Perinatol..

[19] Jin W.Y., Lin S.L., Hou R.L., Chen X.Y., Han T., Jin Y. (2016). Associations between maternal lipid profile and pregnancy complications and perinatal outcomes: a population-based study from China. BMC Pregnancy Childbirth.

[20] Song X., Wang C., Wang T., Zhang S., Qin J. (2023). Obesity and risk of gestational diabetes mellitus: a two-sample Mendelian randomization study. Diabetes Res. Clin. Pract..

[21] Shao H., Xu C., Zhang C., Li L., Wu P., Chen Z. (2025). Genetic insights into lipid traits and lipid-modifying drug targets in pregnancy complications: a two-sample Mendelian randomization study. Int. J. Womens Health.

[22] Zhang Y., Lan X., Cai C., Li R., Gao Y., Yang L. (2021). Associations between maternal lipid profiles and pregnancy complications: a prospective population-based study. Am. J. Perinatol.

[23] You Y., Hu H., Cao C., Han Y., Tang J., Zhao W. (2023). Association between the triglyceride to high-density lipoprotein cholesterol ratio and the risk of gestational diabetes mellitus: a second analysis based on data from a prospective cohort study. Front Endocrinol..

[24] Tang M., Zhao Q., Yi K., Wu Y., Xiang Y., Cui S. (2022). Association between four nontraditional lipids and ischemic stroke: a cohort study in shanghai, China. Lipids Health Dis..

[25] Pirillo A., Catapano A.L. (2024). Remnant cholesterol: a reliable prognostic marker?. Eur. J. Prev. Cardiol..

[26] Pan D., Xu L., Zhang L.X., Shi D.Z., Guo M. (2024). Associations between remnant cholesterol levels and mortality in patients with diabetes. World J. Diabetes.

[27] Huh J.H., Roh E., Lee S.J., Ihm S.H., Han K.D., Kang J.G. (2023). Remnant cholesterol is an independent predictor of type 2 diabetes: a nationwide population-based cohort study. Diabetes Care.

[28] Zhang Y., Song K., Bi S., Li M., Yao Z. (2024). Higher remnant cholesterol increases the risk of coronary heart disease and diabetes in postmenopausal women. Front Endocrinol..

[29] Su S., Zhang E., Gao S., Zhang Y., Liu J., Xie S. (2024). Associations of remnant cholesterol in early pregnancy with gestational diabetes mellitus risk: a prospective birth cohort study. Lipids Health Dis..

[30] Wang W., Li N., Wang X., Zhang X., Tu M., Lin L. (2023). Remnant cholesterol is associated with gestational diabetes mellitus: a cohort study. J. Clin. Endocrinol. Metab..

[31] Gao Y., Hu Y., Xiang L. (2023). Remnant cholesterol, but not other cholesterol parameters, is associated with gestational diabetes mellitus in pregnant women: a prospective cohort study. J. Transl Med..

[32] Yu J., Xia X., Lin T., Huang N., Qiu Y., Yang X. (2021). Non-high-density lipoprotein cholesterol and mortality among peritoneal dialysis patients. J. Clin. Lipidol..

[33] Li G., Huang W., Zhang L., Tian Z., Zheng W., Wang T. (2018). A prospective cohort study of early-pregnancy risk factors for gestational diabetes in polycystic ovarian syndrome. Diabetes Metab. Res. Rev..

[34] Cibickova L., Langova K., Schovanek J., Macakova D., Krystynik O., Karasek D. (2022). Pregnancy lipid profile and different lipid patterns of gestational diabetes treated by diet itself. Physiol. Res..

[35] Magnussen E.B., Vatten L.J., Lund-Nilsen T.I., Salvesen K.A., Davey Smith G., Romundstad P.R. (2007). Prepregnancy cardiovascular risk factors as predictors of pre-eclampsia: population based cohort study. BMJ.

[36] Adank M.C., Benschop L., Peterbroers K.R., Smak Gregoor A.M., Kors A.W., Mulder M.T. (2019). Is maternal lipid profile in early pregnancy associated with pregnancy complications and blood pressure in pregnancy and long term postpartum?. Am. J. Obstet. Gynecol..

[37] Ma N., Bai L., Lu Q. (2024). First-trimester triglyceride-glucose index and triglyceride/high-density lipoprotein cholesterol are predictors of gestational diabetes mellitus among the four surrogate biomarkers of insulin resistance. Diabetes Metab. Syndr. Obes. Targets Ther..

[38] Wang D., Xu S., Chen H., Zhong L., Wang Z. (2015). The associations between triglyceride to high-density lipoprotein cholesterol ratios and the risks of gestational diabetes mellitus and large-for-gestational-age infant. Clin. Endocrinol. (Oxf).

[39] Iimura Y., Matsuura M., Yao Z., Ito S., Fujiwara M., Yoshitsugu M. (2015). Lack of predictive power of plasma lipids or lipoproteins for gestational diabetes mellitus in Japanese women. J. Diabetes Investig..

[40] Jiang X., Zhuang J., Juan Y., Zheng X., Zhang H. (2024). Association between remnant cholesterol and the risk of cardiovascular disease in Chinese population. J. Stroke Cerebrovasc. Dis..

[41] Metzger B.E., Gabbe S.G., Persson B., Buchanan T.A., Catalano P.A., International Association of Diabetes and Pregnancy Study Groups Consensus Panel (2010). International association of diabetes and pregnancy study groups recommendations on the diagnosis and classification of hyperglycemia in pregnancy. Diabetes Care.

[42] Hypertension in pregnancy (2013). Report of the American college of obstetricians and gynecologists’ task force on hypertension in pregnancy. Obstet. Gynecol..

[43] MacDonald C.J., Madika A.L., Bonnet F., Fagherazzi G., Lajous M., Boutron-Ruault M.C. (2020). Consumption of cocoa-containing foods and risk of hypertension in French women. Eur. J. Epidemiol..

[44] Xingyan X., Luo S., Lin J., Zhou J., Zheng L., Yang L. (2024). Association between maternal lipid profiles and lipid ratios in early to middle pregnancy as well as their dynamic changes and gestational diabetes mellitus. BMC Pregnancy Childbirth.

[45] Baumfeld Y., Novack L., Wiznitzer A., Sheiner E., Henkin Y., Sherf M. (2015). Pre-conception dyslipidemia is associated with development of preeclampsia and gestational diabetes mellitus. PLoS One.

[46] Wang B., Guo Z., Li H., Zhou Z., Lu H., Ying M. (2022). Non-HDL cholesterol paradox and effect of underlying malnutrition in patients with coronary artery disease: a 41,182 cohort study. Clin. Nutr. Edinb. Scotl.

[47] Chang T.I., Streja E., Ko G.J., Naderi N., Rhee C.M., Kovesdy C.P. (2018). Inverse association between serum non–high-density lipoprotein cholesterol levels and mortality in patients undergoing incident hemodialysis. J. Am. Heart Assoc. Cardiovasc. Cerebrovasc. Dis..

[48] Song Y., Liu J., Zhao K., Gao L., Zhao J. (2021). Cholesterol-induced toxicity: an integrated view of the role of cholesterol in multiple diseases. Cell Metab.

[49] Paul R., Choudhury A., Choudhury S., Mazumder M.K., Borah A. (2016). Cholesterol in pancreatic β-Cell death and dysfunction. Pancreas.

[50] Li B., Liu Y., Zhou X., Chen L., Yan L., Tang X. (2024). Remnant cholesterol is more positively related to diabetes, prediabetes, and insulin resistance than conventional lipid parameters and lipid ratios: a multicenter, large sample survey. J. Diabetes.

[51] Wu Y., Wei Q., Li H., Yang H., Wu Y., Yu Y. (2023). Association of remnant cholesterol with hypertension, type 2 diabetes, and their coexistence: the mediating role of inflammation-related indicators. Lipids Health Dis..

[52] Hu X., Liu Q., Guo X., Wang W., Yu B., Liang B. (2022). The role of remnant cholesterol beyond low-density lipoprotein cholesterol in diabetes mellitus. Cardiovasc. Diabetol..

[53] Ying W., Fu W., Lee Y.S., Olefsky J.M. (2020). The role of macrophages in obesity-associated islet inflammation and β-cell abnormalities. Nat. Rev. Endocrinol..

[54] Shen L., Wang D., Huang Y., Ye L., Zhu C., Zhang S. (2022). Longitudinal trends in lipid profiles during pregnancy: association with gestational diabetes mellitus and longitudinal trends in insulin indices. Front Endocrinol..

[55] Spracklen C.N., Smith C.J., Saftlas A.F., Robinson J.G., Ryckman K.K. (2014). Maternal hyperlipidemia and the risk of preeclampsia: a meta-analysis. Am. J. Epidemiol..

[56] Chalas J., Audibert F., Francoual J., Le Bihan B., Frydman R., Lindenbaum A. (2002). Concentrations of apolipoproteins E, C2, and C3 and lipid profile in preeclampsia. Hypertens. Pregnancy.

[57] Emet T., Üstüner I., Güven S.G., Balık G., Ural Ü.M., Tekin Y.B. (2013). Plasma lipids and lipoproteins during pregnancy and related pregnancy outcomes. Arch. Gynecol. Obstet..

[58] Caruso A., Ferrazzani S., De Carolis S., Lucchese A., Lanzone A., De Santis L. (1999). Gestational hypertension but not pre-eclampsia is associated with insulin resistance syndrome characteristics. Hum. Reprod..

[59] Catarino C., Rebelo I., Belo L., Rocha-Pereira P., Rocha S., Bayer Castro E. (2008). Fetal lipoprotein changes in pre-eclampsia. Acta Obstet. Gynecol. Scand..

[60] Stefanović M., Vukomanović P., Milosavljević M., Kutlešić R., Popović J., Tubić-Pavlović A. (2009). Insulin resistance and c-reactive protein in preeclampsia. Bosn J. Basic Med. Sci..

[61] Barter P.J., Caulfield M., Eriksson M., Grundy S.M., Kastelein J.J.P., Komajda M. (2007). Effects of torcetrapib in patients at high risk for coronary events. N. Engl. J. Med..

[62] Schwartz G.G., Olsson A.G., Abt M., Ballantyne C.M., Barter P.J., Brumm J. (2012). Effects of dalcetrapib in patients with a recent acute coronary syndrome. N. Engl. J. Med..

[63] van der Steeg W.A., Holme I., Boekholdt S.M., Larsen M.L., Lindahl C., Stroes E.S.G. (2008). High-density lipoprotein cholesterol, high-density lipoprotein particle size, and apolipoprotein a-I: significance for cardiovascular risk: the IDEAL and EPIC-Norfolk studies. J. Am. Coll. Cardiol..

[64] Riwanto M., Landmesser U. (2013). High density lipoproteins and endothelial functions: mechanistic insights and alterations in cardiovascular disease1. J. Lipid Res..

[65] Navab M., Anantharamaiah G.M., Reddy S.T., Van Lenten B.J., Ansell B.J., Fogelman A.M. (2006). Mechanisms of disease: proatherogenic HDL—An evolving field. Nat. Clin. Pract. Endocrinol. Metab..

[66] Li J., Lu J., Wang M., Hu W., Jin N., Li X. (2021). Predictive value of second-trimester maternal lipid profiling in early-onset pre-eclampsia: a prospective cohort study and nomogram. Front Med..

[67] Kharb S., Gulati N., Singh V., Singh G.P. (1998). Lipid peroxidation andVitamin E level s in preeclampsia. Gynecol. Obstet. Invest.

[68] Barrett H.L., Dekker Nitert M., McIntyre H.D., Callaway L.K. (2014). Maternal lipids in pre-eclampsia: innocent bystander or culprit?. Hypertens. Pregnancy.

[69] Enquobahrie D.A., Williams M.A., Butler C.L., Frederick I.O., Miller R.S., Luthy D.A. (2004). Maternal plasma lipid concentrations in early pregnancy and risk of preeclampsia. Am. J. Hypertens..

[70] Winkler K., Wetzka B., Hoffmann M.M., Friedrich I., Kinner M., Baumstark M.W. (2003). Triglyceride-rich lipoproteins are associated with hypertension in preeclampsia. J. Clin. Endocrinol. Metab..

[71] Herrera E., Lasunción M.A., Polin R.A., Fox W.W., Abman S.H. (2004). Fetal and Neonatal Physiology.

[72] Agarwal V., Gupta B.K., Vishnu A., Mamtatyagi S., Kiran J. (2014). Association of lipid profile and uric acid with pre-eclampsia of third trimester in nullipara women. J. Clin. Diagn. Res..

[73] Ghio A., Bertolotto A., Resi V., Volpe L., Di Cianni G. (2011). Triglyceride metabolism in pregnancy. Adv. Clin. Chem..

[74] Adiga U., D’souza V., Kamath A., Mangalore N. (2007). Antioxidant activity and lipid peroxidation in preeclampsia. J. Chin Med. Assoc..

[75] Uzun H., Benian A., Madazli R., Topçuoğlu M.A., Aydin S., Albayrak M. (2005). Circulating oxidized low-density lipoprotein and paraoxonase activity in preeclampsia. Gynecol. Obstet. Invest.

[76] Koçyigit Y., Atamer Y., Atamer A., Tuzcu A., Akkus Z. (2004). Changes in serum levels of leptin, cytokines and lipoprotein in pre-eclamptic and normotensive pregnant women. Gynecol. Endocrinol..

[77] Alston M.C., Redman L.M., Sones J.L. (2022). An overview of obesity, cholesterol, and systemic inflammation in preeclampsia. Nutrients.

[78] Li M., Zhang W., Zhang M., Li L., Wang D., Yan G. (2024). Nonlinear relationship between untraditional lipid parameters and the risk of prediabetes: a large retrospective study based on Chinese adults. Cardiovasc. Diabetol..

[79] Yang H., Han R., Wang Z. (2023). Third-child fertility intention and its socioeconomic factors among women aged 20–34 years in China. BMC Public Health.

