# Supplementary material for: The association between early pregnancy maternal lipid indicators with gestational diabetes mellitus and pre-eclampsia
Source: J Lipid Res. 2025 Nov 11;66(12):100942. doi: 10.1016/j.jlr.2025.100942 (PMC12732318; doi:10.1016/j.jlr.2025.100942)
Supplement: Supplementary Material [file mmc1.docx]

**Attachment information**

**Contents**

**[Fig. S1](#_Toc206533857)** [Flow chart showing the process of participant's recruitment 2](#_Toc206533857)

**[Table S1](#_Toc206533858)** [Incidence and 95% confidence intervals of outcomes across quartiles of lipid profiles and lipid-derived indicators 3](#_Toc206533858)

**[Table S2](#_Toc206533859)** [Subgroup analysis of the association between RC and outcomes 4](#_Toc206533859)

**[Table S3](#_Toc206533860)** [Subgroup analysis of the association between TG and outcomes 5](#_Toc206533860)

**[Table S4](#_Toc206533861)** [Subgroup analysis of the association between TC and outcomes 6](#_Toc206533861)

**[Table S5](#_Toc206533862)** [Subgroup analysis of the association between HDL-C and PE 7](#_Toc206533862)

**[Table S6](#_Toc206533863)** [Subgroup analysis of the association between Non-HDL-C and outcomes 8](#_Toc206533863)

**[Table S7](#_Toc206533864)** [Subgroup analysis of the association between TG/HDL-C ratio and outcomes 9](#_Toc206533864)


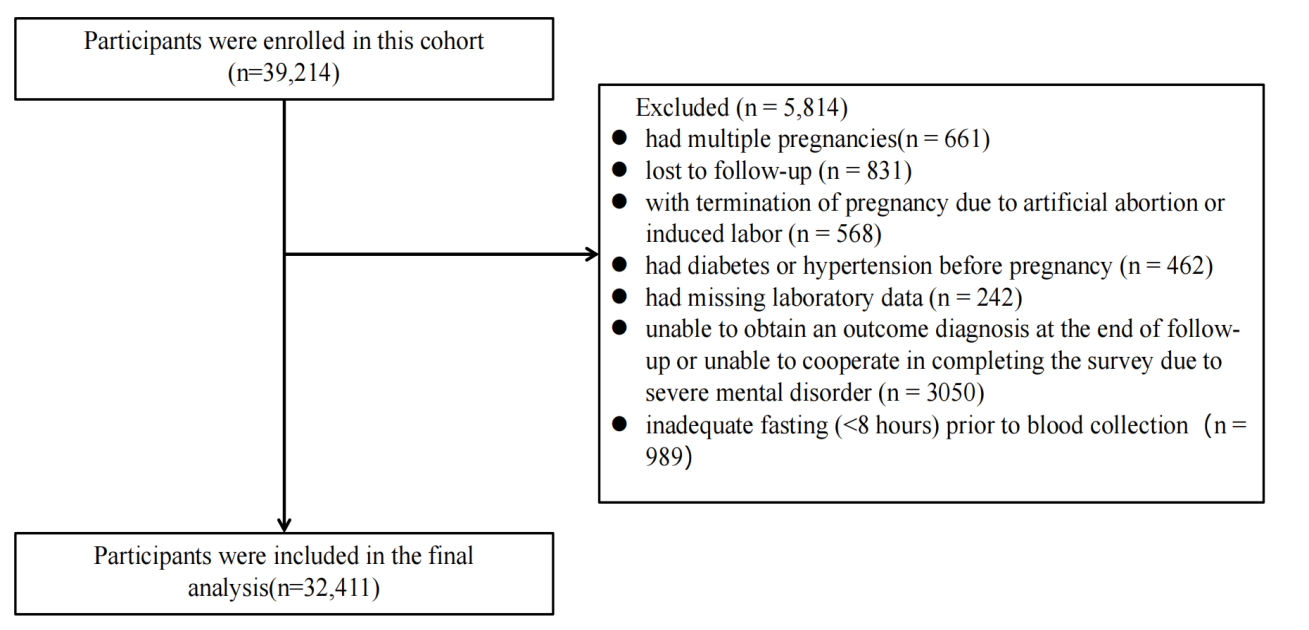


**Fig. S1** Flow chart showing the process of participant’s recruitment

**Table S1** Incidence and 95% confidence intervals of outcomes across quartiles of lipid profiles and lipid-derived indicators

| Variables | GDM % (95%CI) | PE % (95%CI) | GDM/PE % (95%CI) |
| --- | --- | --- | --- |
|  |  |  |  |
| Overall | 15.37 (14.98-15.77) | 1.86 (1.71-2.00) | 16.89 (16.48-17.30) |
| TG quartile |  |  |  |
| Q1 (<2.76) | 12.02 (11.31-12.73) | 1.38 (1.13-1.64) | 13.21 (12.47-13.95) |
| Q2 (2.76-3.50) | 13.16 (12.43-13.90) | 1.51 (1.25-1.78) | 14.43 (13.66-15.20) |
| Q3 (3.50-4.22) | 16.28 (15.47-17.08) | 2.48 (2.15-2.82) | 18.35 (17.51-19.20) |
| Q4 (≥4.22) | 19.95 (19.09-20.82) | 2.04 (1.73-2.35) | 21.48 (20.59-22.37) |
| TC quartile |  |  |  |
| Q1 (<5.68) | 16.63 (15.81-17.44) | 1.89 (1.59-2.18) | 18.39 (17.54-19.24) |
| Q2 (5.68-6.41) | 13.53 (12.72-14.34) | 1.77 (1.46-2.09) | 15.23 (14.38-16.09) |
| Q3 (6.41-7.14) | 15.84 (15.10-16.58) | 2.04 (1.75-2.32) | 17.24 (16.48-18.01) |
| Q4 (≥7.14) | 15.15 (14.37-15.93) | 1.69 (1.41-1.97) | 16.40 (15.60-17.21) |
| HDL-C quartile |  |  |  |
| Q1 (<1.54) | 16.87 (16.05-17.70) | 1.82 (1.52-2.11) | 18.46 (17.61-19.32) |
| Q2 (1.54-1.72) | 14.85 (13.99-15.71) | 2.29 (1.92-2.65) | 16.77 (15.86-17.67) |
| Q3 (1.72-1.94) | 15.24 (14.52-15.95) | 2.10 (1.81-2.38) | 16.95 (16.21-17.70) |
| Q4 (≥1.94) | 14.49 (13.73-15.26) | 1.27 (1.03-1.51) | 15.38 (14.60-16.17) |
| LDL-C quartile |  |  |  |
| Q1 (<3.15) | 17.08 (16.26-17.91) | 1.90 (1.60-2.20) | 18.86 (18.01-19.72) |
| Q2 (3.15-3.63) | 13.95 (13.11-14.80) | 1.55 (1.25-1.85) | 15.51 (14.63-16.38) |
| Q3 (3.63-4.14) | 15.79 (15.06-16.51) | 2.37 (2.06-2.67) | 17.37 (16.62-18.13) |
| Q4 (≥4.14) | 14.33 (13.56-15.09) | 1.45 (1.19-1.71) | 15.48 (14.70-16.27) |
| RC quartile |  |  |  |
| Q1 (<0.81) | 14.07 (13.31-14.83) | 1.78 (1.49-2.07) | 15.74 (14.94-16.53) |
| Q2 (0.81-1.02) | 14.14 (13.37-14.91) | 1.45 (1.18-1.72) | 15.45 (14.65-16.25) |
| Q3 (1.02-1.19) | 15.96 (15.17-16.76) | 2.06 (1.75-2.36) | 17.51 (16.68-18.33) |
| Q4 (≥1.19) | 17.21 (16.40-18.02) | 2.12 (1.81-2.42) | 18.75 (17.92-19.59) |
| Non-HDL-C quartile |  |  |  |
| Q1 (<4.02) | 16.42 (15.61-17.23) | 1.89 (1.59-2.19) | 18.18 (17.34-19.03) |
| Q2 (4.02-4.69) | 14.12 (13.32-14.91) | 1.66 (1.37-1.95) | 15.56 (14.74-16.39) |
| Q3 (4.69-5.30) | 16.12 (15.35-16.89) | 2.04 (1.75-2.34) | 17.73 (16.94-18.53) |
| Q4 (≥5.30) | 14.68 (13.91-15.45) | 1.81 (1.52-2.09) | 15.92 (15.12-16.71) |
| TG/HDL-C ratio quartile |  |  |  |
| Q1 (<1.53) | 12.41 (11.69-13.13) | 1.24 (1.00-1.48) | 13.47 (12.73-14.21) |
| Q2 (1.53-2.07) | 13.98 (13.22-14.73) | 1.42 (1.16-1.68) | 15.22 (14.44-16.00) |
| Q3 (2.07-2.57) | 14.93 (14.15-15.71) | 2.43 (2.09-2.76) | 16.89 (16.08-17.71) |
| Q4 (≥2.57) | 20.18 (19.30-21.05) | 2.35 (2.02-2.68) | 21.99 (21.09-22.89) |

Abbreviations: GDM, gestational diabetes mellitus; PE, pre-eclampsia; RC, remnant cholesterol; TG, triglyceride; TC, total cholesterol; HDL-C, high density lipoprotein cholesterol; LDL-C, low density lipoprotein cholesterol; Non-HDL-C, non-high density lipoprotein cholesterol; *CI*, confidence interval.

**Table S2** Subgroup analysis of the association between RC and outcomes

| Characteristics | RC Quartiles (mmol/L) | | | | *P* value | *P*-interaction |
| --- | --- | --- | --- | --- | --- | --- |
|  | Q1 (<0.81) | Q2 (0.81-1.02) | Q3 (1.02-1.19) | Q4 (≥1.19) |  |  |
| GDM | | | | | | |
| Maternal age (years) |  |  |  |  |  | **0.009** |
| <35 | 1.00 (Ref) | 1.135 (1.001-1.288) | 1.218 (1.041-1.424) | 1.386 (1.146-1.676) | **0.010** |  |
| ≥35 | 1.00 (Ref) | 1.001 (0.819-1.223) | 1.300 (1.011-1.672) | 1.267 (0.933-1.721) | 0.073 |  |
| Residence location |  |  |  |  |  | **0.024** |
| Urban areas | 1.00 (Ref) | 1.186 (1.038-1.356) | 1.261 (1.068-1.489) | 1.475 (1.204-1.806) | **0.003** |  |
| Rural areas | 1.00 (Ref) | 0.974 (0.817-1.161) | 1.329 (1.067-1.655) | 1.282 (0.981-1.674) | **0.006** |  |
| Educational level |  |  |  |  |  | **<0.001** |
| Junior high school or below | 1.00 (Ref) | 0.751 (0.498-1.132) | 0.858 (0.513-1.436) | 1.687 (0.935-3.044) | **0.005** |  |
| Senior middle school | 1.00 (Ref) | 1.561 (1.276-1.909) | 1.542 (1.194-1.991) | 0.866 (0.621-1.208) | **<0.001** |  |
| College | 1.00 (Ref) | 1.007 (0.860-1.180) | 1.297 (1.070-1.572) | 1.650 (1.310-2.078) | **<0.001** |  |
| Master or above | 1.00 (Ref) | 0.895 (0.688-1.163) | 1.112 (0.797-1.549) | 1.337 (0.897-1.991) | 0.091 |  |
| Pre—pregnancy maternal BMI |  |  |  |  |  | **<0.001** |
| Normal weight | 1.00 (Ref) | 1.151 (1.011-1.311) | 1.383 (1.179-1.622) | 1.468 (1.209-1.781) | **<0.001** |  |
| Underweight | 1.00 (Ref) | 1.225 (0.862-1.742) | 0.991 (0.627-1.564) | 0.802 (0.470-1.367) | 0.149 |  |
| Overweight | 1.00 (Ref) | 0.850 (0.655-1.104) | 1.373 (0.987-1.910) | 1.573 (1.044-2.369) | **0.003** |  |
| Obesity | 1.00 (Ref) | 1.749 (0.975-3.136) | 2.485 (1.041-5.934) | 4.075 (1.197-13.876) | 0.120 |  |
| GDM/PE | | | | | | |
| Maternal age (years) |  |  |  |  |  | **0.036** |
| <35 | 1.00 (Ref) | 1.095 (0.969-1.238) | 1.183 (1.017-1.377) | 1.404 (1.168-1.688) | **0.003** |  |
| ≥35 | 1.00 (Ref) | 1.003 (0.828-1.216) | 1.228 (0.963-1.566) | 1.172 (0.871-1.577) | 0.223 |  |
| Residence location |  |  |  |  |  | **0.025** |
| Urban areas | 1.00 (Ref) | 1.167 (1.025-1.329) | 1.244 (1.058-1.463) | 1.522 (1.249-1.855) | **<0.001** |  |
| Rural areas | 1.00 (Ref) | 0.951 (0.804-1.124) | 1.229 (0.997-1.516) | 1.171 (0.908-1.512) | **0.029** |  |
| Educational level |  |  |  |  |  | **<0.001** |
| Junior high school or below | 1.00 (Ref) | 0.644 (0.441-0.94) | 0.831 (0.515-1.341) | 1.601 (0.920-2.787) | **<0.001** |  |
| Senior middle school | 1.00 (Ref) | 1.410 (1.164-1.708) | 1.321 (1.035-1.686) | 0.796 (0.581-1.092) | **<0.001** |  |
| College | 1.00 (Ref) | 1.028 (0.881-1.200) | 1.344 (1.113-1.622) | 1.774 (1.415-2.224) | **<0.001** |  |
| Master or above | 1.00 (Ref) | 0.913 (0.707-1.180) | 1.053 (0.762-1.457) | 1.281 (0.868-1.889) | 0.174 |  |
| Pre—pregnancy maternal BMI |  |  |  |  |  | **<0.001** |
| Normal weight | 1.00 (Ref) | 1.099 (0.969-1.247) | 1.290 (1.105-1.506) | 1.393 (1.155-1.681) | **0.002** |  |
| Underweight | 1.00 (Ref) | 1.326 (0.948-1.856) | 1.026 (0.660-1.595) | 0.911 (0.545-1.525) | 0.103 |  |
| Overweight | 1.00 (Ref) | 0.848 (0.660-1.089) | 1.300 (0.947-1.784) | 1.462 (0.985-2.170) | **0.006** |  |
| Obesity | 1.00 (Ref) | 1.885 (1.082-3.284) | 3.583 (1.516-8.473) | 16.643 (4.657-59.476) | **<0.001** |  |

In the multivariate models, confounding factors such as maternal age, residence location, education, ethnicity, monthly household income, maternal pre-pregnancy body mass index, drinking before pregnancy, smoking before pregnancy, drinking in early pregnancy, smoking in early pregnancy, parity, family history of diabetes, family history of hyperglycemia and other lipid indicators (TG, TC, LDL-C, HDL-C, TG/HDLC ratio and Non-HDL-C) were included unless the variable was used as a subgroup variable. Abbreviations: GDM, gestational diabetes mellitus; PE, pre-eclampsia; RC, remnant cholesterol; TG, triglyceride; TC, total cholesterol; HDL-C, high density lipoprotein cholesterol; LDL-C, low density lipoprotein cholesterol; Non-HDL-C, non-high density lipoprotein cholesterol; *OR*, odds ratio; *CI*, confidence interval.

**Table S3** Subgroup analysis of the association between TG and outcomes

| Characteristics | TG Quartiles (mmol/L) | | | | *P* value | *P*-interaction |
| --- | --- | --- | --- | --- | --- | --- |
|  | Q1 (<2.76) | Q2 (2.76-3.50) | Q3 (3.50-4.22) | Q4 (≥4.22) |  |  |
| GDM | | | | | | |
| Maternal age (years) |  |  |  |  |  | **0.015** |
| <35 | 1.00 (Ref) | 1.307 (1.133-1.507) | 1.832 (1.521-2.208) | 2.040 (1.629-2.554) | **<0.001** |  |
| ≥35 | 1.00 (Ref) | 0.723 (0.576-0.909) | 0.825 (0.613-1.110) | 1.111 (0.780-1.581) | **<0.001** |  |
| Pre—pregnancy maternal BMI |  |  |  |  |  | **<0.001** |
| Normal weight | 1.00 (Ref) | 1.026 (0.887-1.187) | 1.297 (1.074-1.566) | 1.410 (1.123-1.772) | **0.002** |  |
| Underweight | 1.00 (Ref) | 2.229 (1.524-3.260) | 5.126 (3.101-8.472) | 11.651 (6.180-21.965) | **<0.001** |  |
| Overweight | 1.00 (Ref) | 0.841 (0.618-1.144) | 1.010 (0.670-1.522) | 1.192 (0.732-1.939) | 0.180 |  |
| Obesity | 1.00 (Ref) | 2.364 (1.192-4.689) | 1.910 (0.757-4.819) | 1.027 (0.361-2.926) | **0.005** |  |
| Parity |  |  |  |  |  | **0.017** |
| Primipara | 1.00 (Ref) | 1.282 (1.022-1.609) | 2.077 (1.538-2.806) | 3.227 (2.242-4.645) | **<0.001** |  |
| Multipara | 1.00 (Ref) | 1.055 (0.915-1.215) | 1.291 (1.073-1.553) | 1.321 (1.058-1.650) | **0.013** |  |
| PE | | | | | | |
| Educational level |  |  |  |  |  | **0.009** |
| Junior high school or below | 1.00 (Ref) | 0.251 (0.118-0.535) | 0.599 (0.222-1.616) | 0.838 (0.225-3.118) | **0.002** |  |
| Senior middle school | 1.00 (Ref) | 1.339 (0.730-2.455) | 0.790 (0.368-1.698) | 0.563 (0.224-1.417) | 0.058 |  |
| College | 1.00 (Ref) | 1.747 (0.924-3.304) | 2.093 (0.931-4.708) | 0.572 (0.216-1.517) | **<0.001** |  |
| Master or above | 1.00 (Ref) | - | 2.767 (0.447-17.150) | 1.357 (0.177-10.369) | 0.208 |  |
| GDM/PE | | | | | | |
| Maternal age (years) |  |  |  |  |  | **0.002** |
| <35 | 1.00 (Ref) | 1.270 (1.106-1.457) | 1.808 (1.510-2.164) | 1.885 (1.516-2.344) | **<0.001** |  |
| ≥35 | 1.00 (Ref) | 0.756 (0.606-0.942) | 0.914 (0.686-1.218) | 1.123 (0.797-1.580) | **0.002** |  |
| Pre—pregnancy maternal BMI |  |  |  |  |  | **<0.001** |
| Normal weight | 1.00 (Ref) | 1.049 (0.910-1.209) | 1.344 (1.120-1.614) | 1.317 (1.054-1.644) | **0.002** |  |
| Underweight | 1.00 (Ref) | 1.990 (1.388-2.853) | 4.766 (2.957-7.682) | 10.858 (5.919-19.918) | **<0.001** |  |
| Overweight | 1.00 (Ref) | 0.875 (0.651-1.175) | 1.136 (0.768-1.681) | 1.348 (0.845-2.149) | 0.080 |  |
| Obesity | 1.00 (Ref) | 2.340 (1.225-4.469) | 1.551 (0.639-3.768) | 0.896 (0.327-2.454) | **0.002** |  |
| Parity |  |  |  |  |  | **0.004** |
| Primipara | 1.00 (Ref) | 1.270 (1.016-1.586) | 2.069 (1.545-2.771) | 2.760 (1.937-3.932) | **<0.001** |  |
| Multipara | 1.00 (Ref) | 1.043 (0.910-1.195) | 1.315 (1.101-1.571) | 1.294 (1.044-1.604) | **0.003** |  |

In the multivariate models, confounding factors such as maternal age, residence location, education, ethnicity, monthly household income, maternal pre-pregnancy body mass index, drinking before pregnancy, smoking before pregnancy, drinking in early pregnancy, smoking in early pregnancy, parity, family history of diabetes, family history of hyperglycemia and other lipid indicators (RC, TC, LDL-C, HDL-C, TG/HDLC ratio and Non-HDL-C) were included unless the variable was used as a subgroup variable. “-”: the sample size is too small to calculate. Abbreviations: GDM, gestational diabetes mellitus; PE, pre-eclampsia; RC, remnant cholesterol; TG, triglyceride; TC, total cholesterol; HDL-C, high density lipoprotein cholesterol; LDL-C, low density lipoprotein cholesterol; Non-HDL-C, non-high density lipoprotein cholesterol; *OR*, odds ratio; *CI*, confidence interval.

**Table S4** Subgroup analysis of the association between TC and outcomes

| Characteristics | TC Quartiles (mmol/L) | | | | *P* value | *P*-interaction |
| --- | --- | --- | --- | --- | --- | --- |
|  | Q1 (<5.68) | Q2 (5.68-6.41) | Q3 (6.41-7.14) | Q4 (≥7.14) |  |  |
| GDM | | | | | | |
| Maternal age (years) |  |  |  |  |  | **<0.001** |
| <35 | 1.00 (Ref) | 0.874 (0.724-1.054) | 1.157 (0.888-1.509) | 1.603 (1.134-2.264) | **<0.001** |  |
| ≥35 | 1.00 (Ref) | 1.542 (1.158-2.052) | 1.919 (1.275-2.889) | 1.728 (1.020-2.929) | **0.007** |  |
| Pre—pregnancy maternal BMI |  |  |  |  |  | **0.001** |
| Normal weight | 1.00 (Ref) | 1.023 (0.843-1.241) | 1.414 (1.079-1.853) | 1.687 (1.190-2.391) | **0.001** |  |
| Underweight | 1.00 (Ref) | 1.576 (0.912-2.725) | 1.126 (0.525-2.415) | 1.487 (0.579-3.821) | 0.100 |  |
| Overweight | 1.00 (Ref) | 0.849 (0.600-1.202) | 1.339 (0.781-2.294) | 1.411 (0.686-2.902) | 0.090 |  |
| Obesity | 1.00 (Ref) | 5.064 (2.167-11.830) | 3.302 (0.944-11.543) | 27.549 (2.441-310.854) | **<0.001** |  |
| Parity |  |  |  |  |  | **0.014** |
| Primipara | 1.00 (Ref) | 1.269 (0.950-1.694) | 1.908 (1.259-2.891) | 1.748 (1.008-3.029) | **0.008** |  |
| Multipara | 1.00 (Ref) | 0.939 (0.779-1.133) | 1.164 (0.894-1.516) | 1.621 (1.152-2.279) | **<0.001** |  |
| GDM/PE | | | | | | |
| Maternal age (years) |  |  |  |  |  | **<0.001** |
| <35 | 1.00 (Ref) | 0.900 (0.752-1.078) | 1.056 (0.818-1.363) | 1.484 (1.063-2.071) | **<0.001** |  |
| ≥35 | 1.00 (Ref) | 1.609 (1.220-2.123) | 2.168 (1.457-3.227) | 2.095 (1.254-3.500) | **0.001** |  |
| Pre—pregnancy maternal BMI |  |  |  |  |  | **<0.001** |
| Normal weight | 1.00 (Ref) | 1.034 (0.857-1.247) | 1.407 (1.082-1.828) | 1.778 (1.268-2.494) | **<0.001** |  |
| Underweight | 1.00 (Ref) | 1.195 (0.711-2.009) | 0.770 (0.371-1.597) | 1.055 (0.426-2.614) | 0.106 |  |
| Overweight | 1.00 (Ref) | 1.052 (0.753-1.470) | 1.285 (0.765-2.160) | 1.331 (0.665-2.665) | 0.758 |  |
| Obesity | 1.00 (Ref) | 3.469 (1.572-7.652) | 1.373 (0.415-4.543) | 6.190 (0.692-55.366) | **0.001** |  |
| Parity |  |  |  |  |  | **0.030** |
| Primipara | 1.00 (Ref) | 1.281 (0.968-1.694) | 1.861 (1.246-2.781) | 1.686 (0.990-2.870) | **0.009** |  |
| Multipara | 1.00 (Ref) | 0.988 (0.826-1.183) | 1.141 (0.884-1.471) | 1.686 (1.212-2.344) | **<0.001** |  |

In the multivariate models, confounding factors such as maternal age, residence location, education, ethnicity, monthly household income, maternal pre-pregnancy body mass index, drinking before pregnancy, smoking before pregnancy, drinking in early pregnancy, smoking in early pregnancy, parity, family history of diabetes, family history of hyperglycemia and other lipid indicators (TG, RC, LDL-C, HDL-C, TG/HDLC ratio and Non-HDL-C) were included unless the variable was used as a subgroup variable. Abbreviations: GDM, gestational diabetes mellitus; PE, pre-eclampsia; RC, remnant cholesterol; TG, triglyceride; TC, total cholesterol; HDL-C, high density lipoprotein cholesterol; LDL-C, low density lipoprotein cholesterol; Non-HDL-C, non-high density lipoprotein cholesterol; *OR*, odds ratio; *CI*, confidence interval.

**Table S5** Subgroup analysis of the association between HDL-C and PE

| Characteristics | HDL-C Quartiles (mmol/L) | | | | *P* value | *P*-interaction |
| --- | --- | --- | --- | --- | --- | --- |
|  | Q1 (<1.54) | Q2 (1.54-1.72) | Q3 (1.72-1.94) | Q4 (≥1.94) |  |  |
| Educational level |  |  |  |  |  | **0.001** |
| Junior high school or below | 1.00 (Ref) | 2.925 (1.485-5.762) | 4.753 (2.095-10.784) | 1.651 (0.496-5.491) | **<0.001** |  |
| Senior middle school | 1.00 (Ref) | 1.276 (0.814-2.001) | 0.946 (0.564-1.586) | 0.492 (0.238-1.018) | **0.016** |  |
| College | 1.00 (Ref) | 2.732 (1.641-4.547) | 1.830 (0.988-3.391) | 2.265 (1.036-4.953) | **0.001** |  |
| Master or above | 1.00 (Ref) | 1.568 (0.570-4.313) | 3.344 (1.166-9.590) | 10.297 (2.909-36.452) | **0.001** |  |
| Family history of hyperglycemia |  |  |  |  |  | **0.014** |
| Yes | 1.00 (Ref) | - | - | - | - |  |
| No | 1.00 (Ref) | 1.467 (1.113-1.935) | 1.398 (1.017-1.923) | 1.119 (0.726-1.723) | **0.011** |  |

In the multivariate models, confounding factors such as maternal age, residence location, education, ethnicity, monthly household income, maternal pre-pregnancy body mass index, drinking before pregnancy, smoking before pregnancy, drinking in early pregnancy, smoking in early pregnancy, parity, family history of diabetes, family history of hyperglycemia and other lipid indicators (TG, TC, LDL-C, RC, TG/HDLC ratio and Non-HDL-C) were included unless the variable was used as a subgroup variable. “-”: the sample size is too small to calculate. Abbreviations: GDM, gestational diabetes mellitus; PE, pre-eclampsia; RC, remnant cholesterol; TG, triglyceride; TC, total cholesterol; HDL-C, high density lipoprotein cholesterol; LDL-C, low density lipoprotein cholesterol; Non-HDL-C, non-high density lipoprotein cholesterol; *OR*, odds ratio; *CI*, confidence interval.

**Table S6** Subgroup analysis of the association between Non-HDL-C and outcomes

| Characteristics | Non-HDL-C Quartiles (mmol/L) | | | | *P* value | *P*-interaction |
| --- | --- | --- | --- | --- | --- | --- |
|  | Q1 (<4.02) | Q2 (4.02-4.69) | Q3 (4.69-5.30) | Q4 (≥5.30) |  |  |
| GDM | | | | | | |
| Maternal age (years) |  |  |  |  |  | **0.006** |
| <35 | 1.00 (Ref) | 0.709 (0.580-0.867) | 0.527 (0.394-0.706) | 0.330 (0.227-0.481) | **<0.001** |  |
| ≥35 | 1.00 (Ref) | 0.861 (0.639-1.162) | 1.171 (0.742-1.849) | 0.841 (0.468-1.512) | **0.011** |  |
| Educational level |  |  |  |  |  | **0.002** |
| Junior high school or below | 1.00 (Ref) | 1.866 (1.067-3.266) | 2.187 (0.840-5.692) | 2.267 (0.686-7.491) | 0.188 |  |
| Senior middle school | 1.00 (Ref) | 0.660 (0.469-0.930) | 0.604 (0.369-0.989) | 0.384 (0.200-0.739) | **0.014** |  |
| College | 1.00 (Ref) | 0.583 (0.456-0.744) | 0.417 (0.293-0.593) | 0.201 (0.128-0.318) | **<0.001** |  |
| Master or above | 1.00 (Ref) | 0.926 (0.616-1.391) | 1.393 (0.760-2.553) | 1.333 (0.626-2.838) | 0.196 |  |
| Parity |  |  |  |  |  | **0.009** |
| Primipara | 1.00 (Ref) | 0.573 (0.422-0.777) | 0.371 (0.239-0.576) | 0.266 (0.150-0.473) | **<0.001** |  |
| Multipara | 1.00 (Ref) | 0.847 (0.695-1.033) | 0.796 (0.593-1.068) | 0.488 (0.334-0.712) | **<0.001** |  |
| GDM/PE | | | | | | |
| Pre—pregnancy maternal BMI |  |  |  |  |  | **0.001** |
| Normal weight | 1.00 (Ref) | 0.713 (0.583-0.873) | 0.601 (0.451-0.802) | 0.409 (0.284-0.590) | **<0.001** |  |
| Underweight | 1.00 (Ref) | 0.366 (0.212-0.633) | 0.350 (0.162-0.756) | 0.185 (0.069-0.498) | **<0.001** |  |
| Overweight | 1.00 (Ref) | 1.090 (0.761-1.562) | 1.211 (0.682-2.150) | 0.667 (0.310-1.435) | **0.035** |  |
| Obesity | 1.00 (Ref) | 0.155 (0.067-0.362) | 0.039 (0.009-0.170) | - | **<0.001** |  |
| Parity |  |  |  |  |  | **0.044** |
| Primipara | 1.00 (Ref) | 0.648 (0.481-0.872) | 0.432 (0.281-0.664) | 0.269 (0.153-0.471) | **<0.001** |  |
| Multipara | 1.00 (Ref) | 0.801 (0.662-0.969) | 0.768 (0.579-1.020) | 0.498 (0.346-0.718) | **<0.001** |  |

In the multivariate models, confounding factors such as maternal age, residence location, education, ethnicity, monthly household income, maternal pre-pregnancy body mass index, drinking before pregnancy, smoking before pregnancy, drinking in early pregnancy, smoking in early pregnancy, parity, family history of diabetes, family history of hyperglycemia and other lipid indicators (TG, TC, LDL-C, HDL-C, TG/HDLC ratio and RC) were included unless the variable was used as a subgroup variable. “-”: the sample size is too small to calculate. Abbreviations: GDM, gestational diabetes mellitus; PE, pre-eclampsia; RC, remnant cholesterol; TG, triglyceride; TC, total cholesterol; HDL-C, high density lipoprotein cholesterol; LDL-C, low density lipoprotein cholesterol; Non-HDL-C, non-high density lipoprotein cholesterol; *OR*, odds ratio; *CI*, confidence interval.

**Table S7** Subgroup analysis of the association between TG/HDL-C ratio and outcomes

| Characteristics | TG/HDL-C ratio Quartiles | | | | *P* value | *P*-interaction |
| --- | --- | --- | --- | --- | --- | --- |
|  | Q1 (<1.53) | Q2 (1.53-2.07) | Q3 (2.07-2.57) | Q4 (≥2.57) |  |  |
| GDM | | | | | | |
| Educational level |  |  |  |  |  | **<0.001** |
| Junior high school or below | 1.00 (Ref) | 0.717 (0.451-1.140) | 0.300 (0.163-0.555) | 0.133 (0.058-0.302) | **<0.001** |  |
| Senior middle school | 1.00 (Ref) | 1.028 (0.809-1.306) | 1.038 (0.747-1.441) | 1.469 (0.967-2.230) | **0.043** |  |
| College | 1.00 (Ref) | 0.914 (0.754-1.108) | 0.897 (0.695-1.156) | 1.264 (0.923-1.732) | **<0.001** |  |
| Master or above | 1.00 (Ref) | 1.106 (0.813-1.505) | 0.753 (0.494-1.147) | 0.721 (0.425-1.222) | **0.025** |  |
| Pre—pregnancy maternal BMI |  |  |  |  |  | **<0.001** |
| Normal weight | 1.00 (Ref) | 1.112 (0.953-1.298) | 0.964 (0.783-1.188) | 1.176 (0.905-1.528) | **0.009** |  |
| Underweight | 1.00 (Ref) | 0.524 (0.354-0.773) | 0.252 (0.146-0.436) | 0.303 (0.152-0.604) | **<0.001** |  |
| Overweight | 1.00 (Ref) | 0.832 (0.598-1.158) | 0.875 (0.569-1.346) | 0.910 (0.532-1.554) | 0.670 |  |
| Obesity | 1.00 (Ref) | 0.480 (0.223-1.035) | 0.410 (0.158-1.065) | 0.421 (0.127-1.401) | 0.252 |  |
| Parity |  |  |  |  |  | **0.003** |
| Primipara | 1.00 (Ref) | 0.658 (0.516-0.839) | 0.561 (0.403-0.780) | 0.631 (0.418-0.954) | **0.001** |  |
| Multipara | 1.00 (Ref) | 1.117 (0.960-1.299) | 0.932 (0.762-1.138) | 1.158 (0.900-1.490) | **0.001** |  |
| PE | | | | | | |
| Maternal age (years) |  |  |  |  |  | **<0.001** |
| <35 | 1.00 (Ref) | 1.211 (0.750-1.953) | 2.572 (1.421-4.654) | 3.575 (1.712-7.465) | **<0.001** |  |
| ≥35 | 1.00 (Ref) | 0.843 (0.461-1.542) | 1.063 (0.483-2.339) | 0.959 (0.352-2.608) | 0.795 |  |
| Educational level |  |  |  |  |  | **0.001** |
| Junior high school or below | 1.00 (Ref) | 3.439 (1.562-7.571) | 2.101 (0.713-6.192) | 1.388 (0.315-6.121) | **0.005** |  |
| Senior middle school | 1.00 (Ref) | 0.852 (0.429-1.692) | 3.184 (1.428-7.101) | 3.674 (1.317-10.253) | **<0.001** |  |
| College | 1.00 (Ref) | 0.927 (0.486-1.767) | 0.944 (0.399-2.233) | 2.394 (0.862-6.649) | **0.008** |  |
| Master or above | 1.00 (Ref) | 0.201 (0.028-1.426) | 1.408 (0.191-10.369) | 1.245 (0.125-12.369) | **0.006** |  |
| Parity |  |  |  |  |  | **<0.001** |
| Primipara | 1.00 (Ref) | 2.205 (0.808-6.013) | 6.555 (2.013-21.340) | 9.012 (2.286-35.524) | **0.003** |  |
| Multipara | 1.00 (Ref) | 1.079 (0.720-1.617) | 1.601 (0.949-2.702) | 1.894 (0.965-3.717) | 0.119 |  |

In the multivariate models, confounding factors such as maternal age, residence location, education, ethnicity, monthly household income, maternal pre-pregnancy body mass index, drinking before pregnancy, smoking before pregnancy, drinking in early pregnancy, smoking in early pregnancy, parity, family history of diabetes, family history of hyperglycemia and other lipid indicators (TG, TC, LDL-C, HDL-C, RC and Non-HDL-C) were included unless the variable was used as a subgroup variable. Abbreviations: GDM, gestational diabetes mellitus; PE, pre-eclampsia; RC, remnant cholesterol; TG, triglyceride; TC, total cholesterol; HDL-C, high density lipoprotein cholesterol; LDL-C, low density lipoprotein cholesterol; Non-HDL-C, non-high density lipoprotein cholesterol; *OR*, odds ratio; *CI*, confidence interval.

Table S8 Mediation analyses of maternal lipid profiles and lipid-derived indicators in the early pregnancy in the associations between maternal age and outcomes

| Variables | Mediation effect | Direct effect | Mediation proportion | *P value*^a^ |
| --- | --- | --- | --- | --- |
| GDM |  |  |  |  |
| TG | 0.00510 (0.00410, 0.01000) | 0.06780 (0.05691, 0.08000) | 6.998% | **<0.001** |
| RC | 0.00172 (0.00112, 0.00000) | 0.07140 (0.06036, 0.08000) | 2.332% | **<0.001** |
| TG/HDL-C ratio | 0.00352 (0.06945, 0.05850) | 0.06945 (0.05850, 0.08000) | 4.809% | **<0.001** |
| PE |  |  |  |  |
| TG | 0.00037 (0.00008, 0.00000) | 0.00873 (0.00465, 0.01000) | 4.030% | **<0.01** |
| RC | 0.00037 (0.00021, 0.00000) | 0.00880 (0.00473, 0.01000) | 4.070% | **<0.001** |
| Non-HDL-C | 0.00009 (0.00001, 0.00000) | 0.00904 (0.00497, 0.01000) | 0.957% | **<0.05** |
| TG/HDL-C ratio | 0.00034 (0.00014, 0.00000) | 0.00880 (0.00476, 0.01000) | 3.764% | **<0.001** |
| GDM/PE |  |  |  |  |
| TG | 0.00527 (0.00423, 0.01000) | 0.07444 (0.06320, 0.09000) | 6.617% | **<0.001** |
| RC | 0.00193 (0.00126, 0.00000) | 0.07802 (0.06665, 0.09000) | 2.393% | **<0.001** |
| TG/HDL-C ratio | 0.00378 (0.00287, 0.00000) | 0.07601 (0.06471, 0.09000) | 4.728% | **<0.001** |

^a^Adjusted for maternal age, residence location, education, ethnicity, monthly household income, maternal pre-pregnancy body mass index, drinking before pregnancy, smoking before pregnancy, drinking in early pregnancy, smoking in early pregnancy, parity, family history of diabetes, family history of hyperglycemia and other lipid profiles and lipid-derived indicators (TG, TC, LDL-C, HDL-C, RC, TG/HDL-C ratio and Non-HDL-C) except for the mediation variables. Abbreviations: GDM, gestational diabetes mellitus; PE, pre-eclampsia; RC, remnant cholesterol; TG, triglyceride; TC, total cholesterol; HDL-C, high density lipoprotein cholesterol; LDL-C, low density lipoprotein cholesterol; Non-HDL-C, non-high density lipoprotein cholesterol.
